# Selective disruption of TLR2-MyD88 interaction inhibits inflammation and attenuates Alzheimer’s pathology

**DOI:** 10.1172/JCI96209

**Published:** 2018-07-10

**Authors:** Suresh B. Rangasamy, Malabendu Jana, Avik Roy, Grant T. Corbett, Madhuchhanda Kundu, Sujyoti Chandra, Susanta Mondal, Sridevi Dasarathi, Elliott J. Mufson, Rama K. Mishra, Chi-Hao Luan, David A. Bennett, Kalipada Pahan

**Affiliations:** 1 Department of Neurological Sciences, Rush University Medical Center, Chicago, Illinois, USA.; 2 Barrow Neurological Institute, Phoenix, Arizona, USA.; 3 Medicinal and Synthetic Chemistry Core, Center for Molecular Innovation and Drug Discovery, Northwestern University, Evanston, Illinois, USA.; 4 High Throughput Analysis Laboratory and Department of Molecular Biosciences, Northwestern University, Evanston, Illinois, USA.; 5 Rush Alzheimer’s Disease Center, Rush University Medical Center, Chicago, Illinois, USA.; 6 Division of Research and Development, Jesse Brown Veterans Affairs Medical Center, Chicago, Illinois, USA.

**Keywords:** Inflammation, Neuroscience, Alzheimer’s disease, Autoimmune diseases, Innate immunity

## Abstract

Induction of TLR2 activation depends on its association with the adapter protein MyD88. We have found that TLR2 and MyD88 levels are elevated in the hippocampus and cortex of patients with Alzheimer’s disease (AD) and in a 5XFAD mouse model of AD. Since there is no specific inhibitor of TLR2, to target induced TLR2 from a therapeutic angle, we engineered a peptide corresponding to the TLR2-interacting domain of MyD88 (TIDM) that binds to the BB loop of only TLR2, and not other TLRs. Interestingly, WT TIDM peptide inhibited microglial activation induced by fibrillar Aβ1-42 and lipoteichoic acid, but not 1-methyl-4-phenylpyridinium, dsRNA, bacterial lipopolysaccharide, flagellin, or CpG DNA. After intranasal administration, WT TIDM peptide reached the hippocampus, reduced hippocampal glial activation, lowered Aβ burden, attenuated neuronal apoptosis, and improved memory and learning in 5XFAD mice. However, WT TIDM peptide was not effective in 5XFAD mice lacking TLR2. In addition to its effects in 5XFAD mice, WT TIDM peptide also suppressed the disease process in mice with experimental allergic encephalomyelitis and collagen-induced arthritis. Therefore, selective targeting of the activated status of 1 component of the innate immune system by WT TIDM peptide may be beneficial in AD as well as other disorders in which TLR2/MyD88 signaling plays a role in disease pathogenesis.

## Graphical abstract

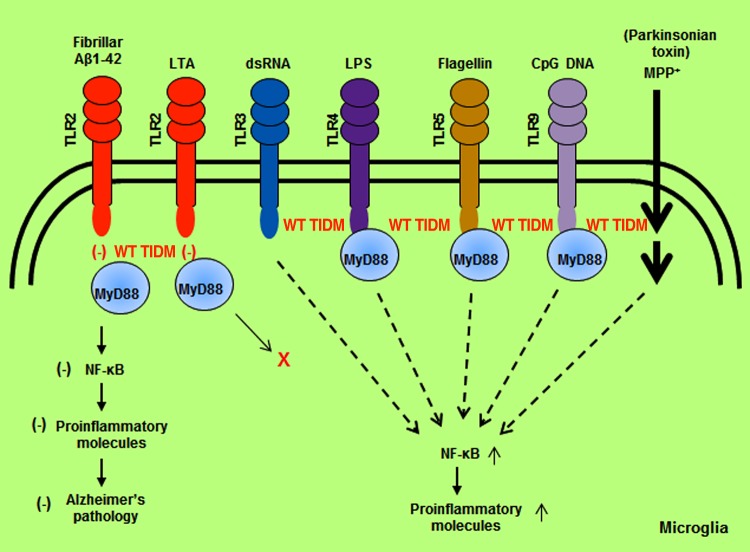

## Introduction

Alzheimer’s disease (AD) is the most common human neurodegenerative disorder that leads to memory loss. It is widely believed that AD is a multifactorial disorder affected by a mix of genetic, environmental, and lifestyle factors (1–3). Neuropathologically, AD is characterized by the presence of senile plaques and neurofibrillary tangles (NFTs) (4–6). Several studies (7–13) also suggest that glial activation and associated inflammation play an important role in the disease pathogenesis and that regulation of neuroinflammation may have therapeutic efficacy in attenuating neurodegeneration in AD.

TLRs serve as important links between innate and adaptive immunity primarily by responding to bacteria, bacterial products, viruses, viral products, and flagellin (14, 15) . Currently, 11 different TLRs have been reported to exist in humans, and all the major CNS cell types are known to express TLRs (15, 16). However, microglia are the only cells in the CNS that express nearly all the TLRs known to date (16). Aside from TLR3, every TLR requires MyD88 for downstream signaling (14, 15). We (17) and others (18, 19) have shown that fibrillar Aβ peptides require TLR2 for microglial inflammation. Here, we demonstrated that TLR2 and MyD88 levels increased in vivo in the frontal cortex and hippocampus of patients with AD and 5XFAD mice. Since no option is available for specific targeting of induced TLR2, we designed a peptide corresponding to the TLR2-interacting domain of MyD88 (TIDM) that specifically inhibited induced TLR2 signaling and fibrillar Aβ–mediated microglial inflammation without modulating dsRNA-, bacterial LPS–, flagellin-, 1-methyl-4-phenylpyridinium– (MPP^+^-), or CpG DNA–mediated microglial activation. Moreover, intranasal administration of TIDM peptide resulted in a reduction in hippocampal microglial activation, lowering of the Aβ load, suppression of neuronal apoptosis, and improvement in memory and learning in 5XFAD mice, highlighting the therapeutic promise of the TIDM peptide in AD.

## Results

### Upregulation of TLR2 in AD.

**Figure 1:**
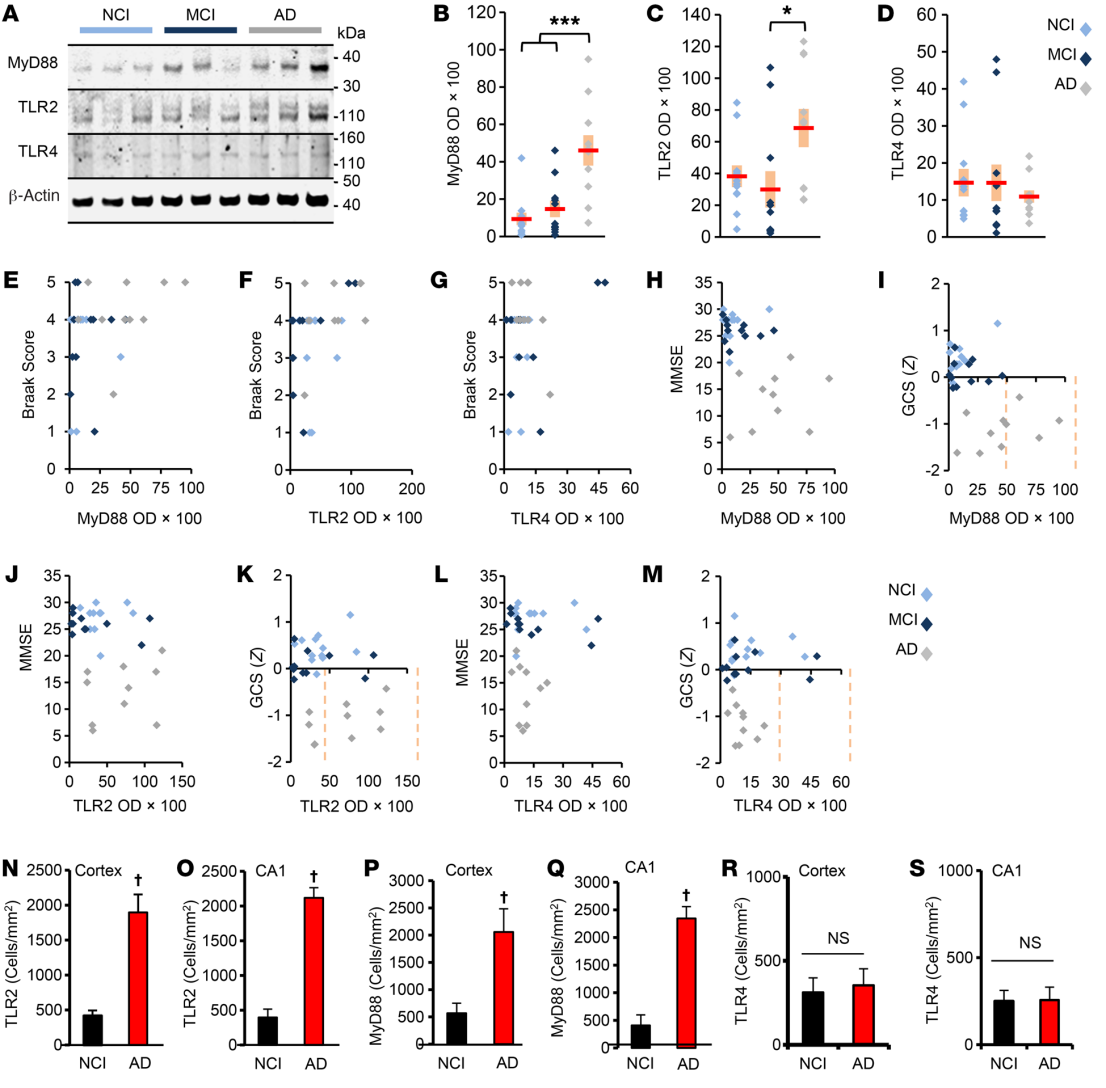
Monitoring TLR2, TLR4, and MyD88 levels in the CNS of individuals clinically diagnosed with NCI, MCI, or AD. (**A**) PFC homogenates (25 μg) from NCI (light blue), MCI (dark blue), and AD (gray) individuals were immunoblotted for TLR2, TLR4, and MyD88. Actin was used to normalize the signals obtained by densitometric measurement (ImageJ). Coomassie was used to verify protein loading. Twelve NCI, eleven MCI, and ten AD samples were run in three independent experiments. (**B**) MyD88 levels were significantly elevated in AD subjects relative to levels in both NCI and MCI (***P < 0.001; Kruskal-Wallis test) subjects. (**C**) TLR2 levels were significantly higher in AD compared with MCI subjects. *P < 0.05; Kruskal-Wallis test. (**D**) TLR4 levels did not differ significantly across the 3 groups. (**E**) MyD88 (0.371, *P* = 0.033) and (**F**) TLR2 (0.463, *P* = 0.007) were positively correlated with the Braak score by Kruskal-Wallis test. (**G**) No such correlation was found between TLR4 (–0.012, *P* = 0.947) and the Braak score. (**H**) MyD88 was negatively correlated with MMSE scores (–0.538, *P* = 0.001) and the (**I**) GCS index (–0.475, *P* = –0.005). However, the negative correlation was not significant for TLR2 with (**J**) the MMSE (–0.278, *P* = 0.117) or (**K**) the GCS (–0.177, *P* = 0.326). TLR4 was also not negatively correlated with (**L**) the MMSE (–0.173, *P* = 0.336) or (**M**) the GCS (0.047, *P* = 0.794). Statistical significance was determined by Spearman’s rank-order test in **G**–**M**. Hippocampal sections of NCI and AD brains were double labeled with Iba-1 (microglia) and TLR2, TLR4, or MyD88. Cells positive for TLR2 (**N**, cortex; **O**, CA1), MyD88 (**P**, cortex; **Q**, CA1), and TLR4 (**R**, cortex; **S**, CA1) were counted in 2 sections (2 images/slide) of each of 4 different cases. ^†^*P* < 0.001 versus NCI; 2-sample *t* test. Data represent the mean ± SEM.

To investigate the role of TLR2 in the pathogenesis of AD, we monitored the level of TLR2 by immunoblot analysis of prefrontal cortex (PFC) (Brodmann area 9) cells from 33 subjects who died with AD dementia (*n* = 10) or mild cognitive impairment (MCI) (*n* = 11) and from age-matched individuals with no cognitive impairment (NCI) (*n* = 12) (Supplemental Table 1; supplemental material available online with this article; https://doi.org/10.1172/JCI96209DS1). We found no significant differences across the groups in terms of age, sex, postmortem interval, brain weight, and Braak scores (Supplemental Table 1). For comparison, we included TLR4. Since all the TLRs except TLR3 use MyD88, we also investigated MyD88. We found that the levels of both TLR2 and MyD88 in PFC were significantly altered among the groups, with AD subjects expressing higher levels of TLR2 and MyD88 relative to levels in the NCI and MCI subjects (Figure 1, A–C and Supplemental Table 2). In contrast, TLR4 levels did not significantly differ across the groups (Figure 1, A and D; Supplemental Table 2). The Spearman rank-order correlation showed that both TLR2 and MyD88 levels in PFC were positively correlated with Braak staging (Figure 1, E and F, and Supplemental Table 2). On the other hand, we found no relationship between TLR4 levels and Braak score (Figure 1G and Supplemental Table 2). Importantly, MyD88 was also negatively correlated with the mini–mental state examination (MMSE) and global cognitive *Z* score (GCS) (Figure 1, H–M, and Supplemental Table 2).

To confirm these findings, we performed double-label immunofluorescence analysis of hippocampal sections. As expected, Iba-1 (a microglial marker) levels were higher in the cortex and hippocampus of AD cells as compared with levels in NCI cells (Supplemental Figure 1, A–E). As with the Western blot results, we detected higher levels of TLR2 (Supplemental Figure 1A and Figure 1, N and O) and MyD88 (Supplemental Figure 1B and Figure 1, P and Q) in the cortex and hippocampus of AD brain compared with levels in NCI brain. Again, there was no difference in TLR4 expression (Supplemental Figure 1C and Figure 1, R and S).

### Upregulation of TLR2 in 5XFAD-Tg mice.

Next, we examined the status of TLR2 and MyD88 in the hippocampus of 5XFAD-Tg mice. As with levels in the CNS of AD subjects, we noticed higher levels of TLR2 (Supplemental Figure 2, A and B) and MyD88 (Supplemental Figure 3, A and B) in cortex and different parts of the hippocampus of Tg mice as compared with age-matched non-Tg mice. We also found increased Iba-1 immunoreactivity and colocalization of many Iba-1–positive cells with TLR2 (Supplemental Figure 2B) and MyD88 (Supplemental Figure 3B) in the cortex and hippocampus of Tg mice. Western blot experiments also confirmed the increase in TLR2 (Supplemental Figure 2, C and D) and MyD88 (Supplemental Figure 3, C and D) levels in the hippocampus of Tg mice as compared with levels in non-Tg mice.

### Design of a peptide corresponding to the TIDM for specific targeting of TLR2.

**Figure 2:**
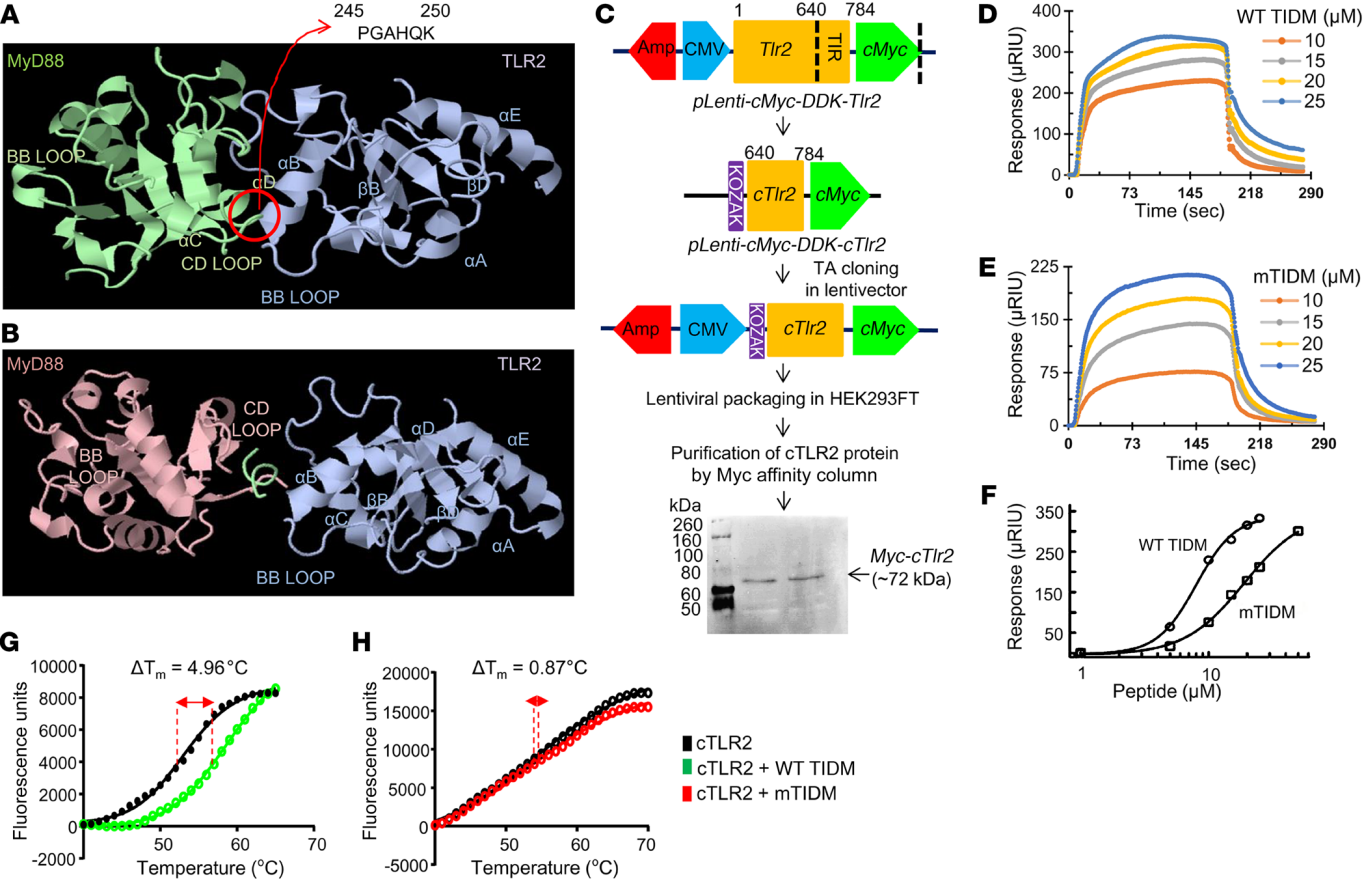
Design of a peptide for disruption of TLR2 and MyD88 interaction. (**A**) A rigid-body, in silico docked pose of mouse TLR2 (blue) and MyD88 (green) (electrostatic energy = –7.750 kcal/mol; desolvation energy = –24.99 kcal/mol; VDW energy = 105.25 kcal/mol; total energy = –22.216 kcal/mol) shows strong interaction between amino acids 245 and 250 of the CD loop of MyD88 and the BB loop of TLR2. Therefore, the peptide corresponding to this domain of MyD88 (TIDM) was used to dissociate the interaction between TLR2 and MyD88. (**B**) TLR2-MyD88 interaction was complexed with the WT TIDM peptide (electrostatic energy = –4.516 kcal/mol; desolvation energy = –24.027 kcal/mol; VDW energy = 16.724 kcal/mol; total energy = –26.871 kcal/mol). (**C**) Generation of a cMyc-tagged cTLR2 recombinant protein. Amp, ampicillin resistance. The in vitro binding affinity of increasing doses of WT TIDM (**D**) and mTIDM (**E**) with cTLR2 was examined using SPR analyses (*n* = 2 replicates/dose in 3 independent experiments). (**F**) Plot of the binding response values versus the concentrations of WT TIDM (circles) and mTIDM (squares) peptides. (**G**) Melting curve of cTLR2 protein (black) alone and with WT TIDM peptides (green). Thermal shift analyses showed a 4.96°C shift of the melting temperature (ΔT_m_) (*n* = 2 replicates/dose in 3 independent experiments). (**H**) Melting curve of cTLR2 protein (black) alone and with mTIDM peptides (red) indicated a ΔT_m_ of 0.87°C (*n* = 2 replicates/dose in 3 independent experiments). μRIU, micro refractive index units. Data represent the mean ± SEM.

Since there is no specific inhibitor of TLR2, for therapeutic purposes, we attempted to target TLR2. After ligand binding, TLR2 functions through MyD88 (14, 15). Therefore, we applied a rigid-body, protein-protein interaction tool to model the interaction between the TLR-interacting domain (TIR) of TLR2 and MyD88. Since the crystal structures of TIRs of murine TLRs were not available, we adopted an silico homology modeling strategy to build 3D structures of TIRs from all different TLRs (Supplemental Figure 4, A–G). Similar to previous findings (20), the docked pose of the MyD88 and TIR complex as derived from our in silico modeling analyses revealed that the BB loop of TLR2 was engaged with the CD loop of MyD88, with a strong van der Waals (VDW) interaction (Figure 2A). Therefore, we designed the following peptides corresponding to the TIDM from the CD loop to disrupt the interaction between TLR2 and MyD88: WT TIDM, drqikiwfqnrrmkwkk^245^PGAHQK^250^ and mutated TIDM (mTIDM), drqikiwfqnrrmkwkk^245^PGWHQD^250^.

We added the Antennapedia homeodomain (lowercase) at the C-terminal of these peptides to facilitate cell permeability. MyD88 segments are in uppercase, and the positions of the mutations are underlined. Interestingly, when the interaction between the TIR of TLR2 and MyD88 was modeled with the WT TIDM peptide, we observed that MyD88 was associated with a certain degree of rotation, leaving its CD loop far removed from the TLR2 BB loop (Figure 2B). According to pyDock analysis, the WT TIDM peptide was found to be docked in the interface of the CD loop, αB helix, and BB loop of the TIR domain of TLR2 (Supplemental Figure 5A). That specific pose of the WT TIDM peptide imposed its VDW surface to be distributed over the BB loop of TLR2 (Supplemental Figure 5A), which was not possible in the case of the mTIDM peptide (Supplemental Figure 5B). We observed a strong electrostatic interaction (2.31 A°) between the NE1 atom of the conserved histidine residue (H82) of the CD loop and the ND atom of the histidine (H4) residue of the WT TIDM peptide (Supplemental Figure 5C). The docked structures of mTIDM with TLR2 clearly indicated that there was a very weak electrostatic interaction (7.26 A°) between the H82 residue of the CD loop and the H4 residue of the mTIDM peptide (Supplemental Figure 5C; right). Moreover, mutation of WT TIDM from lysine to aspartate imposed a negative cloud, which also drove the C-terminal end of mTIDM even further away from the BB loop and more toward the groove of the αB helix (Supplemental Figure 5B). We also assessed the possibility of VDW interaction in that complex by measuring the distance of VDW droplets between 2 close residues of TLR2 and MyD88 (Supplemental Figure 5D). We observed a significant VDW overlap between MyD88 and TLR2 in the absence of WT TIDM. However, when complexed with WT TIDM, the BB loop of TLR2 and the CD loop of MyD88 posed far away from each other, negating any possibility of VDW interaction (Supplemental Figure 5E). To compare the affinity of WT TIDM and mTIDM for TLR2 from another angle, we performed surface plasmon resonance (SPR) analysis. We first cloned and purified the whole TLR2 protein. However, it was not stable, and since the whole TLR2 protein was also not available, we prepared only the C-terminal TIR domain of TLR2 protein (cTLR2) using a viral cloning strategy and purified the protein by Myc affinity column (Figure 2C). Kinetic plots (Figure 2, D and E) clearly demonstrated that increasing doses of both WT TIDM and mTIDM showed binding with cTLR2. However, WT TIDM displayed much stronger affinity than mTIDM toward cTLR2 (Figure 2, D–F). According to the plot of the SPR response at equilibrium versus peptide concentration (Figure 2F), the affinity of WT TIDM (*K_D_* = 8 μM) for cTLR2 was approximately 2.5 times stronger than that of mTIDM (*K_D_*= 19 μM). To further substantiate, we performed a thermal shift assay, which revealed that 10 μM of WT TIDM peptide strongly shifted the melting curve of cTLR2 (Figure 2G). On the other hand, very little shifting was observed for mTIDM (Figure 2H). Together, these results suggest that WT TIDM is a potent small-molecule peptide that strongly interferes with the interaction between TLR2 and MyD88.

**Figure 3:**
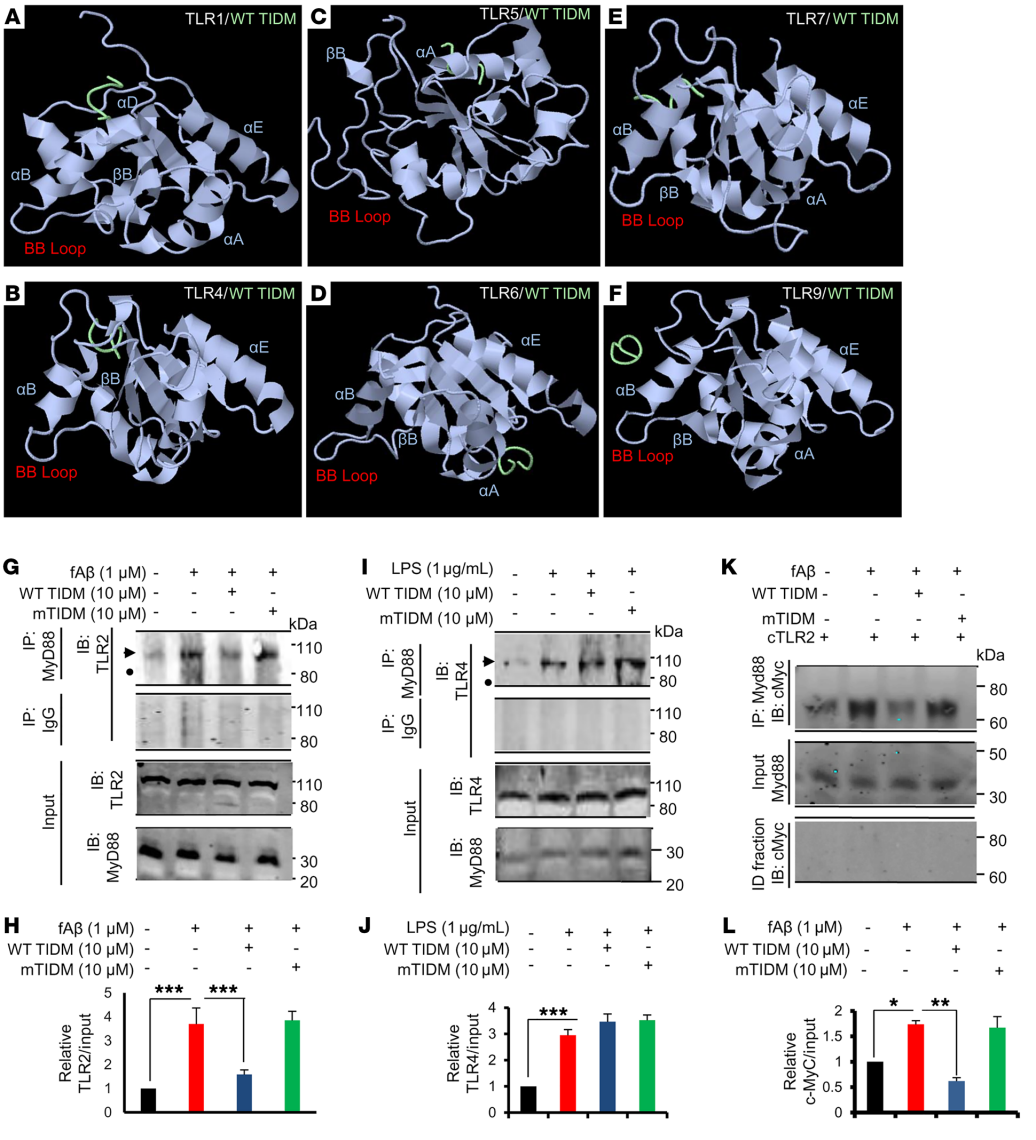
Selective disruption of TLR2 and MyD88 interaction by WT TIDM. In silico analyses of interactions of WT TIDM with TLR1, TLR4, TLR5, TLR6, TLR7, and TLR9. Rigid-body interaction analyses were performed using the pyDock in silico analysis tool. Complexes of TLR1–WT TIDM (**A**), TLR4–WT TIDM (**B**), TLR5–WT TIDM (**C**), TLR6–WT TIDM (**D**), TLR7–WT TIDM (**E**), and TLR9–WT TIDM (**F**) are shown. (**G**) BV-2 microglial cells preincubated with WT TIDM and mTIDM peptides for 1 hour were stimulated with 1 μM fibrillar Aβ1-42 (fAβ) under serum-free conditions. After 1 hour, cellular extracts were immunoprecipitated (IP) with an anti-MyD88 antibody, followed by Western blotting of immunoprecipitates for TLR2. As a control, cellular extracts were immunoprecipitated with normal IgG. Input was also immunoblotted (IB) with TLR2 and MyD88. (**H**) Bands were scanned, and values (TLR2/input) are presented relative to the control (*n* = 2 replicates/condition in 3 independent experiments). ***P < 0.001; 2-sample *t* test. Results were analyzed by 2-sample *t* test. (**I**) BV-2 microglial cells preincubated with WT TIDM and mTIDM peptides for 1 hour were stimulated with LPS under serum-free condition. After 1 hour, cellular extracts were immunoprecipitated with an anti-MyD88 antibody, followed by Western blotting of immunoprecipitates for TLR4. As a control, cellular extracts were immunoprecipitated with normal IgG. Input was also immunoblotted with TLR4 and MyD88. (**J**) Bands were scanned, and values (TLR4/input) are presented relative to the control (*n* = 2 replicates/condition in 3 independent experiments). ***P < 0.001; 2-sample *t* test. (**K**) BV-2 microglial cells were transduced with *pLenti-cMyc-cTlr2* lentivirions, and 48 hours after transduction, cells were treated with WT TIDM and mTIDM for 1 hour, followed by stimulation with fibrillar Aβ1-42. After 1 hour, cellular extracts were immunoprecipitated with anti-MyD88 antibody, followed by Western blotting of immunoprecipitates for cMyc. Immunodepleted (ID) fractions were also immunoblotted for cMyc as a control. (**L**) Bands were scanned and values (cMyc/input) presented relative to the control (*n* = 2 replicates/condition in 3 independent experiments). *P < 0.05 and **P < 0.01; 2-sample *t* test. Data represent the mean ± SEM.

Next, we examined whether WT TIDM had similar affinity for other TLRs. Interestingly, our in silico analyses revealed that the WT TIDM peptide docked far from the BB loop of TLR1 (Figure 3A), TLR4 (Figure 3B), TLR5 (Figure 3C), TLR6 (Figure 3D), TLR7 (Figure 3E), and TLR9 (Figure 3F), suggesting that WT TIDM specifically targets the BB loop of TLR2, but not other TLRs.

Next, we examined whether the WT TIDM peptide could disrupt the physical association between endogenous TLR2 and MyD88. Earlier, we delineated that fibrillar Aβ1-42 activates microglia via TLR2 (17). We performed immunoblot analysis of MyD88 immunoprecipitates with antibodies against TLR2 and found that fibrillar Aβ1-42 treatment increased the association between TLR2 and MyD88 in microglial cells and that this interaction was inhibited by WT TIDM, but not mTIDM, peptide (Figure 3, G and H). Input showed the presence of equal amounts of TLR2 and MyD88 under different treatment conditions (Figure 3G). To understand the specificity, we examined the effect of WT TIDM peptide on the interaction between TLR4 and MyD88. LPS is a prototypical agonist of TLR4. LPS treatment increased the association between TLR4 and MyD88 in microglial cells (Figure 3, I and J) and in contrast to the suppression of TLR2-MyD88 interaction (Figure 3, G and H), WT TIDM peptide had no effect on the interaction between TLR4 and MyD88 (Figure 3, I and J). Next, we examined whether WT TIDM could interfere with the interaction between MyD88 and newly formed Myc-tagged cTLR2. Therefore, microglial cells were transduced with *pLenti-cMyc-cTlr2* lentivirions, and after 48 hours of transduction, cells were treated with fibrillar Aβ1-42 in the presence or absence of WT TIDM or mTIDM for 1 hour. Immunoblot analysis of MyD88 immunoprecipitates with antibodies against cMyc showed that the interaction between newly formed cTLR2 and MyD88 in Aβ1-42–treated microglial cells was inhibited by WT TIDM, but not mTIDM, peptide (Figure 3, K and L).

### TIDM peptide inhibits microglial inflammation induced by fibrillar Aβ1-42 and lipoteichoic acid, but not 1-methyl-4-phenylpyridinium, dsRNA (poly IC), bacterial LPS, flagellin, or CpG DNA.

**Figure 4:**
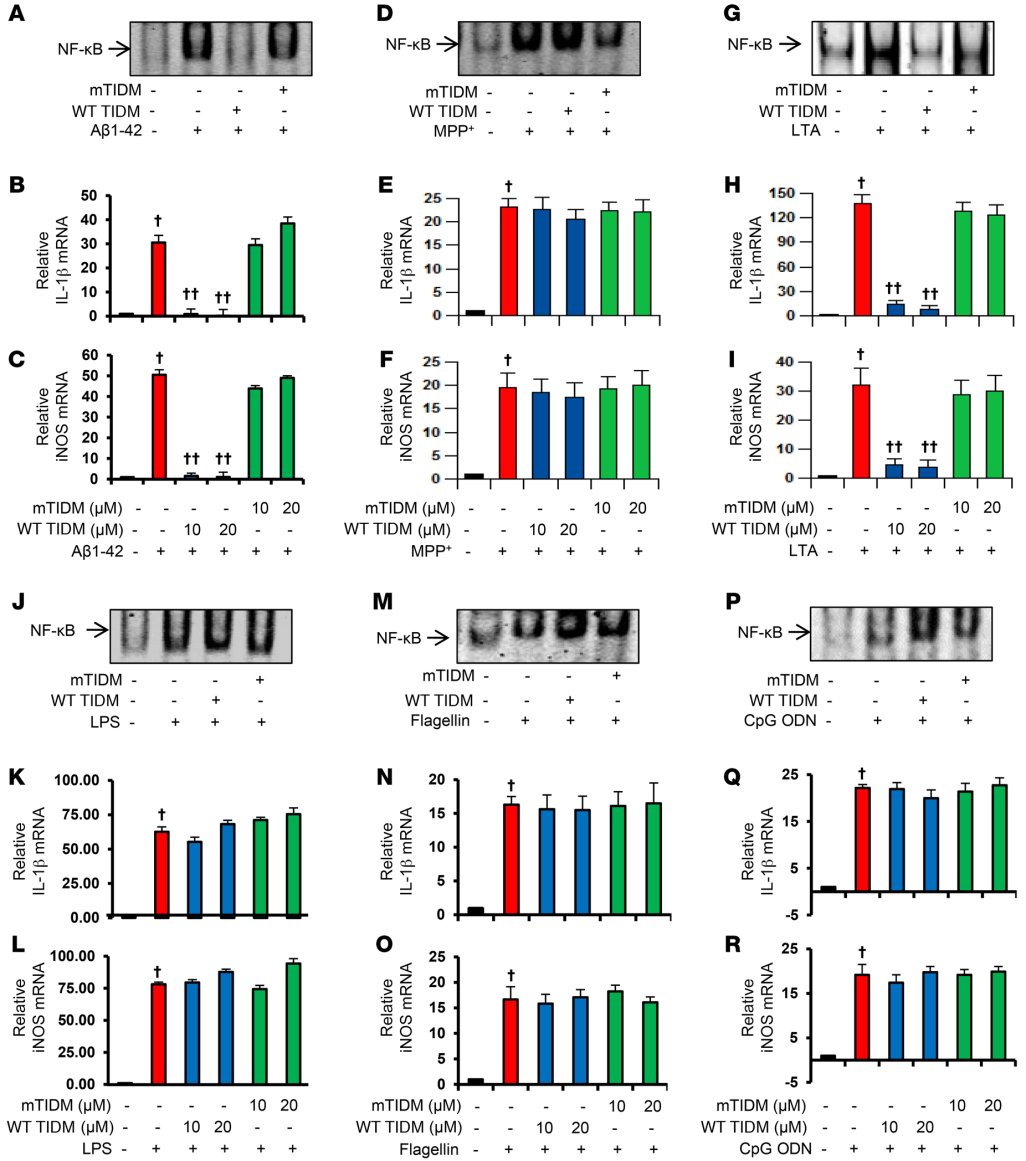
Effect of WT TIDM and mTIDM peptides on the induction of NF-κB activation and expression of proinflammatory molecules in microglial cells. BV-2 microglial cells preincubated with 10 μM WT TIDM or mTIDM peptides for 1 hour were stimulated with (**A**–**C**) 1 μM fibrillar Aβ1-42, (**D–F**) 1 μM MPP^+^, (**G**–**I**) 250 ng/ml LTA, (**J**–**L**) 1 μg/ml LPS, (**M**–**O**) 1 μM flagellin, and (**P**–**R**) 1 μM CpG DNA under serum-free conditions. After 1 hour of stimulation, NF-κB activation was monitored in nuclear extracts by EMSA (**A**, fibrillar Aβ; **D**, MPP^+^; **G**, LTA; **J**, LPS; **M**, flagellin; **P**, CpG DNA). After 4 hours of stimulation, mRNA expression of IL-1β (**B**, **E**, **H**, **K**, **N**, and **Q**) and iNOS (**C**, **F**, **I**, **L**, **O**, and **R**) was monitored by real-time PCR. *n* = 2 replicates/dose in 3 independent experiments. ^†^*P* < 0.001 versus control; ^††^*P* < 0.001 versus stimuli*;* 2-sample *t* test. ODN, oligodeoxynucleotide. Data represent the mean ± SEM.

Microglia expressing different TLRs are activated under various pathological conditions, such as neurodegeneration, inflammation, and viral or bacterial infection. (7, 21). Therefore, we investigated whether TIDM peptide was capable of suppressing microglial activation induced by different stimuli. Microglial cells pretreated with different concentrations of WT TIDM and mTIDM peptides for 1 hour were stimulated with fibrillar Aβ1-42 (an etiological reagent of AD), 1-methyl-4-phenylpyridinium (MPP^+^) (Parkinsonian toxin), lipoteichoic acid (LTA) (agonist of TLR2), poly IC (agonist of TLR3), LPS (agonist of TLR4), flagellin (agonist of TLR5), or CpG DNA (agonist of TLR9). As expected, fibrillar Aβ (Figure 4A), MPP^+^ (Figure 4D), LTA (Figure 4G), poly IC (Supplemental Figure 7A), LPS (Figure 4J), flagellin (Figure 4M), and CpG DNA (Figure 4P) induced the activation of NF-κB in microglial cells. However, WT TIDM peptides inhibited fibrillar Aβ- and LTA-mediated activation of NF-κB (Figure 4, A and G). In contrast, WT TIDM peptides remained unable to suppress the activation of NF-κB in microglial cells induced by MPP^+^ (Figure 4D), poly IC (Supplemental Figure 7A), LPS (Figure 4J), flagellin (Figure 4M), or CpG DNA (Figure 4P). These results were specific, as mTIDM peptides had no effect on the activation of NF-κB induced by any of the stimuli. Activation of the classical NF-κB pathway involves the phosphorylation of IκBα, followed by nuclear translocation of p65 and p50. Therefore, we also investigated the effect of WT TIDM peptide on nuclear translocation of p65 and p50 in activated microglia. As expected, we observed increased nuclear translocation of p65 and p50 in microglial cells in response to fibrillar Aβ1-42 (Supplemental Figure 8, A–C) and LPS (Supplemental Figure 8, D–F). However, WT TIDM peptide treatment inhibited the nuclear translocation of p65 and p50 in microglial cells stimulated with fibrillar Aβ1-42 (Supplemental Figure 8, A–C), but not LPS (Supplemental Figure 8, D–F), indicating the specificity of the WT TIDM peptide. To confirm these results, we also monitored the expression of IL-1β and inducible NO synthase (iNOS), proinflammatory molecules that are driven by NF-κB activation. All stimuli induced the expression of IL-1β and iNOS in microglial cells (Figure 4, B, C, E, F, H, I, K, L, N, O, Q, and R, Supplemental Figure 6, A–F, and Supplemental Figure 7, B–D). Consistent with the effect of WT TIDM on NF-κB activation, WT TIDM peptides inhibited the expression of proinflammatory molecules induced only by fibrillar Aβ (Supplemental Figure 6A and Figure 4, B and C) or LTA (Supplemental Figure 6C and Figure 4, H and I), but not MPP^+^ (Supplemental Figure 6B and Figure 4, E and F), poly IC (Supplemental Figure 7, B–D), LPS (Supplemental Figure 6D and Figure 4, K and L), flagellin (Supplemental Figure 6E and Figure 4, N and O), or CpG DNA (Supplemental Figure 6F and Figure 4, Q and R). These results suggest that the WT TIDM peptide specifically inhibits microglial inflammation induced by agonists of TLR2, but not of other TLRs.

### WT TIDM peptide does not inhibit fibrillar Aβ1-42–induced activation of microglia in the absence of TLR2.

Since WT TIDM peptide disrupted the physical association between TLR2 and MyD88, as a mechanistic proof of principal, we examined the effect of WT TIDM peptide on Aβ1-42–induced activation of *Tlr2^–/–^* microglia. As with BV-2 microglial cells, fibrillar Aβ1-42 peptides strongly induced the activation of NF-κB in primary microglia isolated from WT mice, and this was inhibited by WT TIDM peptide (Supplemental Figure 9A). On the other hand, fibrillar Aβ1-42 peptides weakly induced the DNA-binding activity of NF-κB in *Tlr2^–/–^* microglia (Supplemental Figure 9A). However, in contrast to what was observed in WT microglia, the WT TIDM peptide remained unable to inhibit fibrillar Aβ1-42–induced activation of NF-κB in *Tlr2^–/–^* microglia (Supplemental Figure 9, A and B). To further confirm our findings, we also measured the levels of common proinflammatory cytokines (TNF-α and IL-1β) in supernatants. As with NF-κB activation, the induction of TNF-α and IL-1β production by fibrillar Aβ1-42 was low in *Tlr2^–/–^* microglia as compared with WT microglia (Supplemental Figure 9, C–F). However, the WT TIDM peptide inhibited fibrillar Aβ1-42 peptide–induced production of TNF-α and IL-1β in WT, but not *Tlr2^–/–^*, microglia (Supplemental Figure 9, C–F), suggesting that the WT TIDM peptide needs TLR2 to function.

### Intranasal administration of WT TIDM peptide inhibits inflammation, reduces plaque load, and decreases hyperphosphorylation of tau in the hippocampus of 5XFAD-Tg mice.

**Figure 5:**
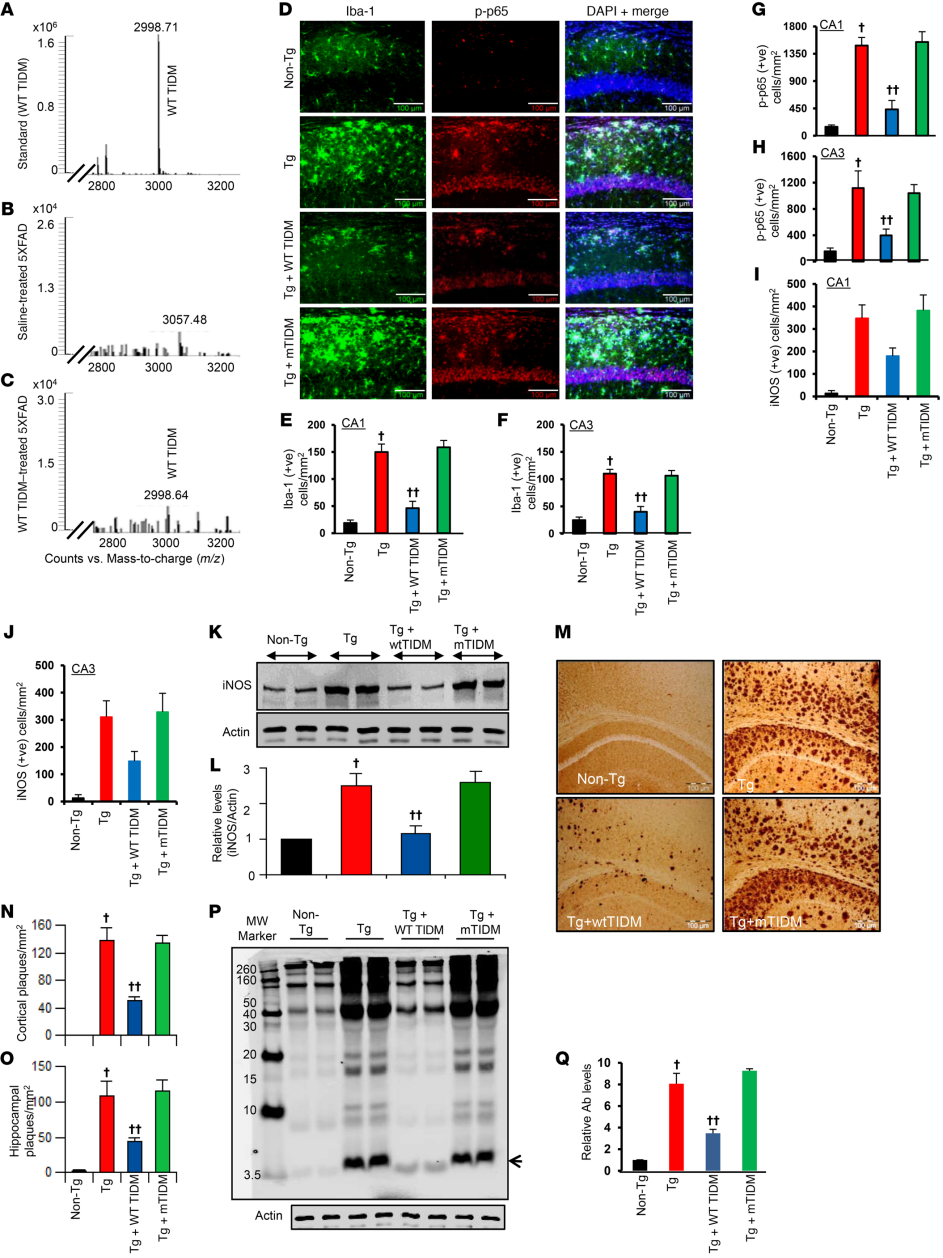
After intranasal delivery, WT TIDM peptide enters into the hippocampus and suppresses glial activation and reduces plaques in the hippocampus of Tg mice. Tg mice (6 months of age) received 1 intranasal dose of WT TIDM peptide (0.1 mg/kg BW). After 60 minutes of treatment, mice were perfused with sterile saline, and hippocampi were homogenized and supernatant analyzed for WT TIDM by ESI-MS (**A**, WT TIDM standard; **B**, untreated 5XFAD-Tg; **C**, WT TIDM–treated 5XFAD-Tg). Tg mice were treated with WT TIDM and mTIDM peptides (0.1 mg/kg body WT/2d) via the intranasal route. After 30 days, hippocampal sections were double labeled for Iba-1 and p-p65 (**D**) and Iba-1 and iNOS (Supplemental Figure 9). Cells positive for Iba-1 (**E**, CA1; **F**, CA3), p-p65 (**G**, CA1; **H**, CA3), and iNOS (**I**, CA1; **J**, CA3) were counted in 2 sections (2 images/slide) from each of 6 different mice (*n* = 6) per group. ^†^*P* < 0.001 versus non-Tg; ^††^*P* < 0.001 versus Tg; 2-sample *t* test. (**K**) Hippocampal extracts from all groups of mice (*n* = 4 per group) were immunoblotted for iNOS. Actin was run as a loading control. Bands were scanned, and values (**L**, iNOS/actin) are presented relative to the non-Tg control. ^†^*P* < 0.001 versus non-Tg; ^††^*P* < 0.001 versus Tg; 2-sample *t* test. (**M**) Hippocampal sections were immunolabeled with 82E1 mAb. Amyloid plaques (**N**, cortex; **O**, hippocampus) were counted in 2 sections (2 images/slide) from each of 6 different mice per group. ^†^*P* < 0.001 versus non-Tg; ^††^*P* < 0.001 versus Tg; 2-sample *t* test. (**P**) Hippocampal extracts (*n* = 4 per group) were analyzed for Aβ by Western blotting using 6E10 mAb. Arrowhead indicates a 4-kDa Aβ band. MW, molecular weight. (**Q**) Bands were scanned, and values (Aβ/actin) are presented relative to the non-Tg control. ^†^*P* < 0.001 versus non-Tg; ^††^*P* < 0.001 versus Tg; 2-sample *t* test. ve, vehicle. Scale bars: 100 μm. Data represent the mean ± SEM.

It is becoming clear that glial inflammation plays an important role in the loss of neurons in AD and other neurodegenerative disorders (7, 9, 22–24). Since the WT TIDM peptide specifically inhibited fibrillar Aβ1-42–mediated microglial activation, we decided to test its therapeutic translatability in 5XFAD-Tg mice. We first determined whether the WT TIDM peptide could enter into the hippocampus. Tg mice were treated with TIDM peptides intranasally, and after 60 minutes of administration, we detected WT TIDM peptide in the hippocampus of Tg mice by electrospray ionization–coupled mass spectrometry (ESI-MS) (Figure 5, A and C). In contrast, the hippocampus of saline-treated Tg mice showed no peak for WT TIDM peptide (Figure 5B). The level of WT TIDM peptide was 23.33 ± 14.14 ng/g brain tissue in the hippocampus of WT TIDM–treated Tg mice compared with nil in the saline-treated Tg mice. By infrared scanning, we also detected TIDM peptide in hippocampus after intranasal treatment (Supplemental Figure 10). Therefore, after intranasal administration, TIDM peptide entered into the hippocampus.

Next, we investigated whether intranasally administered TIDM peptide was capable of modulating NF-κB activation in the hippocampus of Tg mice. As seen by double-label immunofluorescence of hippocampal sections, the levels of Iba-1 and phosphorylated p65 (p-p65) were markedly higher in Tg mice than in non-Tg mice (Figure 5, D–H). However, intranasal treatment of Tg mice with WT TIDM, but not mTIDM, peptides led to the suppression of both Iba-1 and p-p65 in the hippocampus of Tg mice (Figure 5, D–H). This was also confirmed by Western blot analysis of hippocampal tissues (Supplemental Figure 11, A and B). Moreover, activated microglia are known to express iNOS (21, 25). Accordingly, we found that hippocampal microglia of Tg mice were also positive for iNOS (Supplemental Figure 12 and Figure 5, I and J). However, WT TIDM, but not mTIDM, peptide suppressed the expression of iNOS in the hippocampus of Tg mice (Supplemental Figure 12 and Figure 5, I and J). Western blot analysis also confirmed the inhibition of hippocampal iNOS expression by treatment with WT TIDM, but not mTIDM, peptide (Figure 5, K and L).

Amyloid plaque is an important feature of AD pathology, which is modeled in 5XFAD-Tg mice (26, 27). Therefore, we next examined whether WT TIDM treatment is capable of reducing the load of amyloid plaques from the hippocampus of Tg mice. Immunostaining of hippocampal sections with 82E1 mAb (Figure 5, M–O) as well as Western blot analysis of hippocampal tissue with 6E10 mAb (Figure 5, P and Q) and 82E1 mAb (Supplemental Figure 13, A and B) showed markedly higher levels of Aβ peptides in the hippocampus of Tg mice compared with levels in non-Tg mice. Likewise, ELISA of serum (Supplemental Figure 14, A and B), TBS-extracted hippocampal fractions (Supplemental Figure 14, C and D), and TBS plus Triton X-100–extracted hippocampal fractions (Supplemental Figure 14, E and F) also revealed a marked increase in Aβ1-40 and Aβ1-42 in Tg mice compared with non-Tg mice. However, a significant decrease in Aβ was seen with WT TIDM, but not mTIDM, treatment (Supplemental Figure 13, A and B; Supplemental Figure 14, A–F; and Figure 5, M–Q). These results suggest that intranasal administration of WT TIDM is capable of reducing Aβ burden in the hippocampus of 5XFAD mice.

Hyperphosphorylation of tau is another prominent feature of AD pathology (28, 29). It has been shown that hyperphosphorylation of tau at Ser396 occurs in the hippocampus of 5XFAD mice at a much earlier stage than does the appearance of learning and memory impairment (30). Therefore, we examined the effect of TIDM peptide treatment on the status of tau phosphorylation in vivo in the hippocampus of Tg mice. Immunoblot analysis indicated a marked increase in p-tau in hippocampal extracts from Tg mice as compared with levels in extracts from non-Tg mice (Supplemental Figure 15, A and B). However, treatment of Tg mice with WT TIDM, but not mTIDM, peptide led to the suppression of p-tau in the hippocampus, without affecting the total level of tau protein (Supplemental Figure 15, A and B), indicating that WT TIDM peptide treatment is adequate for decreasing p-tau in the hippocampus of Tg mice.

### Reduction in neuronal apoptosis and protection of memory and learning in 5XFAD-Tg mice by intranasal administration of WT TIDM peptide.

**Figure 6:**
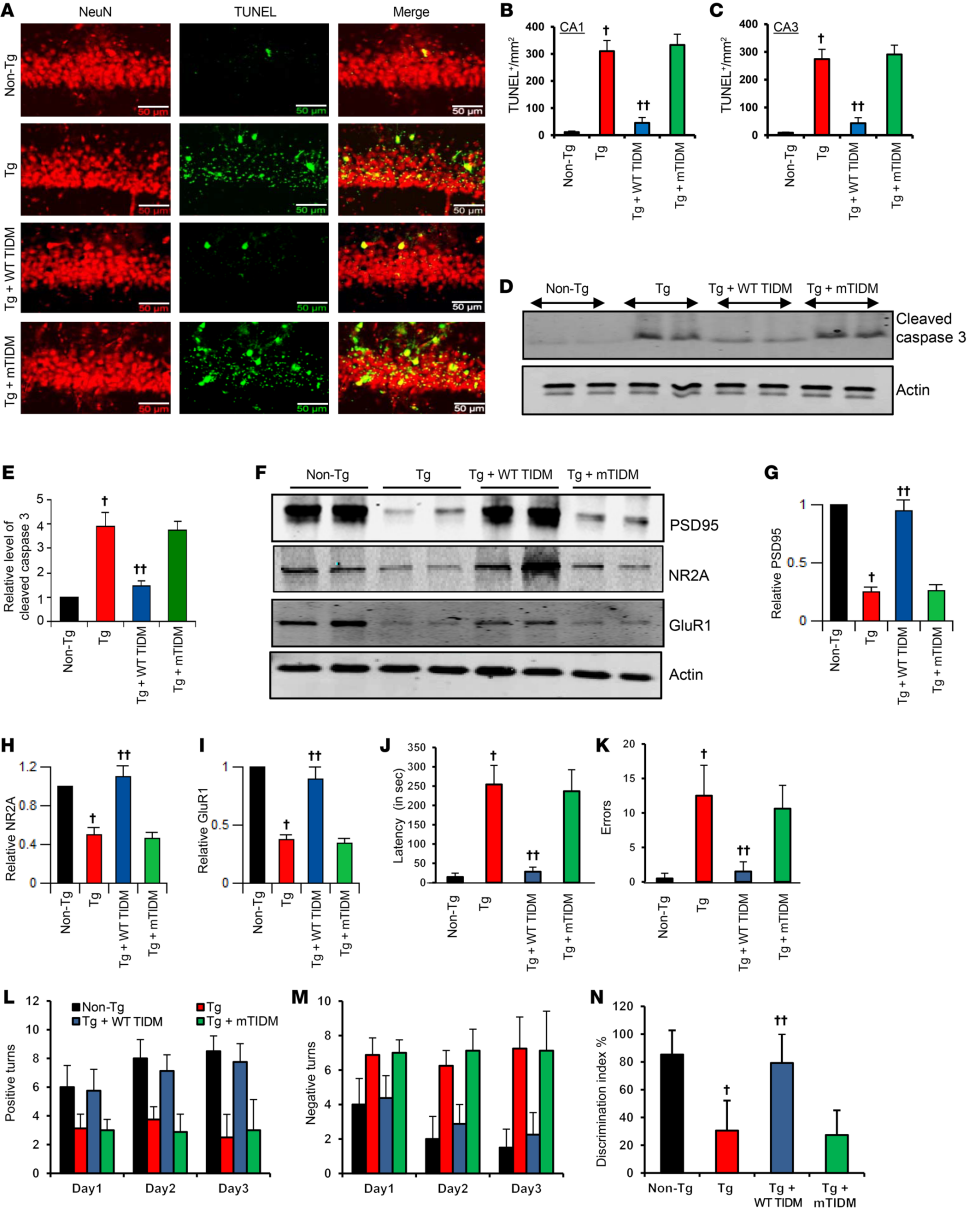
Intranasal delivery of WT TIDM, but not mTIDM, peptide inhibits neuronal apoptosis in vivo in the hippocampus and improves memory and learning in Tg mice. Tg mice (6 months of age) were treated intranasally with WT TIDM and mTIDM peptides (0.1 mg/kg BW/2 d). (**A**) After 30 days of treatment, mice were sacrificed, and hippocampal sections were double labeled for TUNEL and NeuN. Scale bars: 50 μm. TUNEL-positive cells (**B**, CA1; **C**, CA3) were counted in 2 sections (2 images/slide) from each of 6 different mice (*n* = 6) per group. ^†^*P* < 0.001 versus non-Tg; ^††^*P* < 0.001 versus Tg; 2-sample *t* test. (**D**) Hippocampal extracts from all groups of mice (*n* = 4) were immunoblotted for cleaved caspase 3. Actin was run as a loading control. (**E**) Bands were scanned, and the values (cleaved caspase 3/actin) are presented relative to the non-Tg control. Results are expressed as the mean ± SEM of 4 mice per group. ^†^*P* < 0.001 versus non-Tg; ^††^*P* < 0.001 versus Tg*;* 2-sample *t* test. (**F**) Protein levels of PSD95, NR2A, and GluR1 were monitored in hippocampal extracts by Western blot analysis. Bands were scanned, and the values (**G**, PSD95/actin; **H**, NR2A/actin; **I**, GluR1/actin) are presented relative to the non-Tg control. Results are expressed as the mean ± SEM of 4 mice per group. ^†^*P* < 0.001 versus non-Tg; ^††^*P* < 0.001 versus Tg; 2-sample *t* test. Mice were tested using the Barnes maze (**J**, latency; **K**, number of errors made) and T-maze (**L**, number of positive turns; **M**, number of negative turns). (**N**) Short-term memory was also assessed by the NOR test, which is represented by the discrimination index. Eight mice were used in each group, and the results were analyzed by 1-way ANOVA.

Since neuroinflammation may be associated with neuronal apoptosis, we next examined whether WT TIDM peptide treatment was able to reduce neuronal apoptosis in the hippocampus of Tg mice. A number of TUNEL-positive bodies colocalized with NeuN in the hippocampus of Tg mice compared with that seen in non-Tg mice (Figure 6, A–C). However, WT TIDM, but not mTIDM, peptide attenuated neuronal apoptosis in the hippocampus (Figure 6, A–C). This result was confirmed by the detection of cleaved caspase 3. As expected, cleaved caspase 3 levels increased in the hippocampus of Tg mice (Figure 6, D and E). However, treatment of Tg mice with WT TIDM, but not mTIDM, peptide reduced the elevated levels of cleaved caspase 3 in the hippocampus (Figure 6, D and E), suggesting that WT TIDM peptide treatment is capable of decreasing neuronal apoptosis in vivo in the hippocampus of Tg mice. Accordingly, the levels of plasticity-related molecules (PSD95, NR2A, and GluR1) decreased in the hippocampus of Tg mice as compared with levels in non-Tg mice (Figure 6, F–I). However, consistent with the suppression of neuronal apoptosis, treatment of Tg mice with WT TIDM, but not mTIDM, peptide led to significant restoration of PSD95, NR2A, and GluR1 protein levels in vivo in the hippocampus (Figure 6, F–I).

The ultimate objective of neuroprotection in AD is to improve and/or protect memory. Major functions of the hippocampus are to generate and organize long-term memory and spatial learning. Therefore, we examined whether WT TIDM peptide protected memory and learning in Tg mice. As expected, Tg mice took much longer to find the food reward hole and had greater latency (*P* < 0.001 [= 0.0000213]) with higher errors (*P* < 0.001 [= 0.0000251]) in the Barnes maze as compared with non-Tg mice. However, WT TIDM treatment significantly improved the memory function of Tg mice, as shown by their latency (F_3,28_ = 93.153, *P* < 0.001 [= 0.0000112]) (Figure 6J) and number of errors (F_3,28_ = 36.339, *P* < 001 [= 0.0000863]) (Figure 6K). Memory functions of the WT TIDM peptide–treated mice were also better, as demonstrated by their ability to locate the reward hole with less latency (*P* < 0.001 [= 0.0000600]) and fewer errors (*P* < 0.001 [= 0.0000579]) when compared with mTIDM-treated mice. Likewise, on the T-maze test, untreated Tg mice also had fewer positive turns (*P* < 0.001 [= 0.0000440]) and a higher number of negative turns (*P* < 0.001 [= 0.000223]) than did age-matched non-Tg mice (Figure 6, L and M). However, WT TIDM treatment had a significant effect on the number of successful positive turns (F_3,28_ = 31.475, *P* < 0.001 [= 0.0000411]) (Figure 6L) and also resulted in fewer errors (F_3,28_ = 26.653, *P* < 0.001 [= 0.0000235]) (Figure 6M) by Tg mice. Again, WT TIDM–treated mice had a higher number of positive turns (*P* < 0.001 [= 0.0000954]) and fewer negative turns (*P* < 0.001 [= 0.000123]) as compared with mTIDM-treated Tg mice (Figure 6, L and M). We also monitored the short-term memory of Tg mice using the novel object recognition (NOR) test. Tg mice exhibited significant deficits (*P* < 0.001 [= 0.0000149]) in the NOR test as evidenced by the discrimination index (Figure 6N) compared with age-matched non-Tg mice. However, WT TIDM peptide–treated mice showed significant improvement (*P* < 0.001) in short-term memory as compared with either the untreated Tg or mTIDM-treated Tg mice (Figure 6N). On the other hand, the gross motor activity of Tg and non-Tg mice was nearly similar (Supplemental Figure 16). Furthermore, neither the WT TIDM nor the mTIDM peptide modulated the gross motor activity of Tg mice, as evidenced by the number of movements, horizontal activity, rest time, and stereotypy (Supplemental Figure 16, A–D), suggesting that improvement of memory by WT TIDM peptide treatment is not due to alterations in gross motor activity.

### WT TIDM peptide requires TLR2 to reduce plaques and improve memory in 5XFAD-Tg mice.

To confirm that the WT TIDM peptide does in fact require TLR2 to exert its function in vivo, we crossed *Tlr2^–/–^* mice with Tg mice to create 5XFAD mice null for *Tlr2* (*Tlr2^–/–^*-Tg mice). Knockdown of *Tlr2* did not alter the insertion or expression of the 5XFAD transgenes, and vice versa (Supplemental Figure 17A). Six-month-old WT, *Tlr2^–/–^*, Tg, and *Tlr2^–/–^*-Tg mice did not differ significantly with respect to gross BW or wet brain weights (Supplemental Figure 17, B and C). We also did not find any overt phenotypic differences, including diet, fecal boli, social interaction, or agitation across genotypes at this age. Although WT TIDM peptide reduced plaque load and improved spatial learning and memory in Tg mice (Figure 5 and Figure 6), it remained unable to do so in *Tlr2^–/–^*-Tg mice (Supplemental Figure 17, D–G), indicating that WT TIDM peptide is ineffective in the absence of *Tlr2*.

### WT TIDM, but not mTIDM, peptide suppresses the disease process of experimental allergic encephalomyelitis and collagen-induced arthritis in mice.

**Figure 7:**
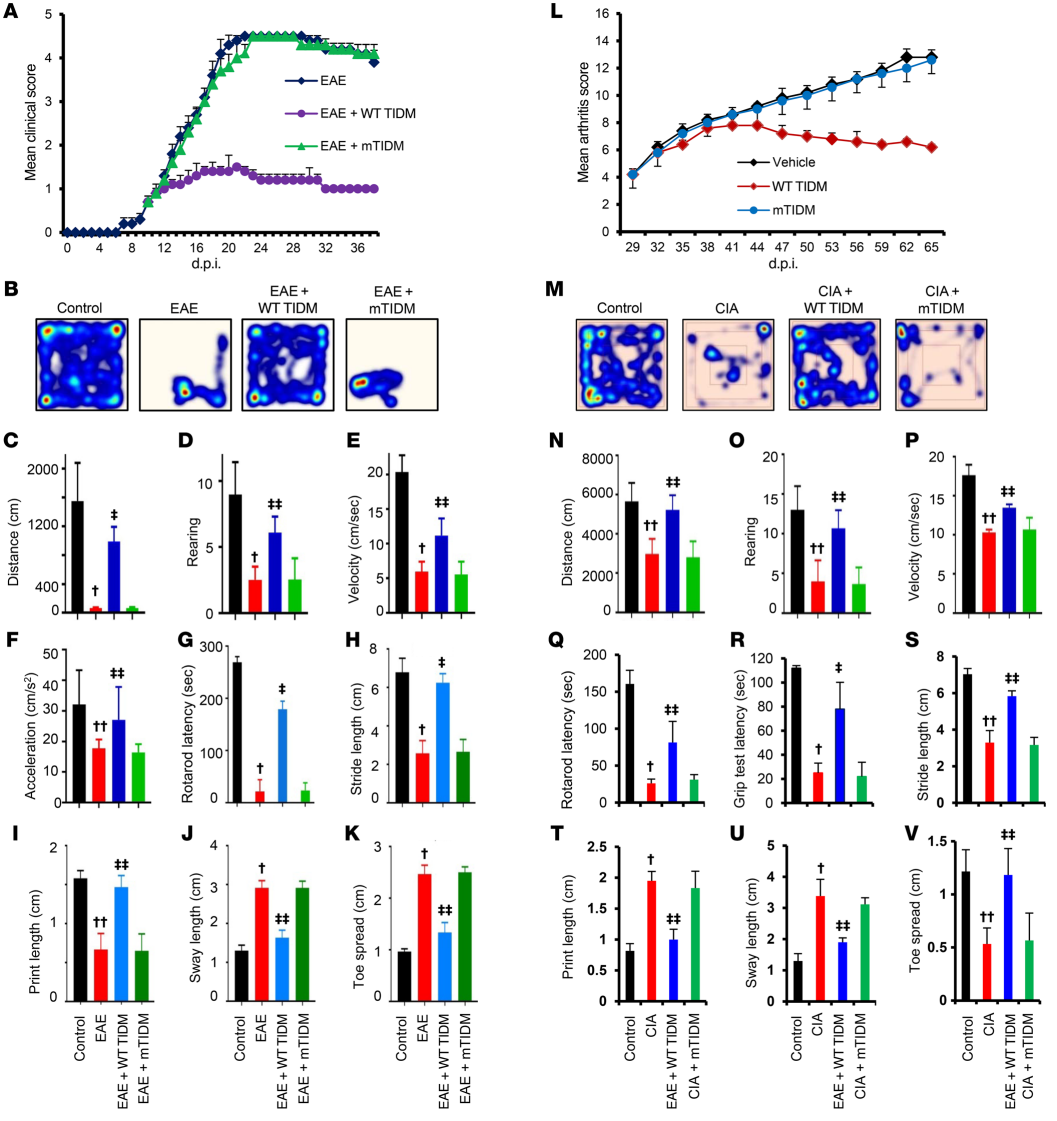
WT TIDM, but not mTIDM, peptide protects mice from EAE and CIA. (**A**) EAE was induced in male C57/BL6 mice by MOG35-55 immunization, and from 10 dpi, mice were treated with intranasal WT TIDM and mTIDM peptides (0.1 mg/kg BW/d). Mice (*n* = 6 per group in 2 independent experiments) were scored daily. As evident by 1-way, repeated-measures ANOVA, the WT TIDM peptide significantly protected mice from EAE (F_2,94_ = 22.59 [>Fc = 3.093]). On day 22 after immunization, general motor activity was assessed using the EthoVision XT 13.0 Open Field Activity System (Noldus) (**B**, heatmap images representing overall motor activity; **C**, distance traveled; **D**, rearing; **E**, velocity; **F**, acceleration) and (**G**) Rotarod testing. Footprint analysis (**H**, stride length; **I**, print length; **J**, sway length; **K**, toe spread) was also performed. (**L**) CIA was induced in male DBA/1J mice by bovine type II collagen immunization, and from 29 dpi, mice were treated with WT TIDM and mTIDM peptides (1 mg/kg BW/d) via intraperitoneal injection. Mice (*n* = 6/group in 2 independent experiments) were scored daily. Repeated-measures, 1-way ANOVA showed that the WT TIDM peptide significantly protected against CIA (F_2,45_ = 4.927 [>Fc = 3.093]). On day 60 after immunization, general motor activity was assessed using the EthoVision system (**M**, heatmap images representing overall motor activity; **N**, distance traveled; **O**, rearing; **P**, velocity), Rotarod testing (**Q**), and grip strength testing (**R**). Footprint analysis (**S**, stride length; **T**, print length; **U**, sway length; **V**, toe spread) was also performed. Six mice (*n* = 6/group) were used in 2 independent experiments. ^†^*P* < 0.001 and ^††^*P* < 0.05 versus control; ^‡^*P* < 0.001 and ^‡‡^*P* < 0.05 versus EAE or CIA by 2-sample *t* test*.* Data represent the mean ± SEM.

As an important member of the innate immune pathways, Myd88-dependent TLR2 signaling plays a significant role in the pathogenesis of a wide variety of infectious and autoimmune disorders (31, 32). Therefore, we examined whether WT TIDM peptide function is limited only to 5XFAD mice or whether it has effects in other disease models as well. Experimental allergic encephalomyelitis (EAE) is a widely used animal model of multiple sclerosis, and a chronic form of EAE is modeled in male C57/BL6 mice upon immunization with MOG35-55. Like its effect in 5XFAD mice, intranasal treatment of EAE mice with WT TIDM peptide strongly inhibited the clinical symptoms of EAE (Figure 7A). When we compared the means between groups with Dunnett’s multiple comparisons analyses, we found that there was a significant difference of means between EAE and EAE plus WT TIDM (adjusted *P* < 0.001). On the other hand, the mTIDM peptide had no effect (Figure 7A), indicating the specificity of the effect. As expected, the induction of EAE reduced locomotor activity in mice, which was evidenced by heatmap analysis (Figure 7B), distance traveled (Figure 7C), rearing (Figure 7D), velocity (Figure 7E), acceleration (Figure 7F), and Rotarod performance (Figure 7G). Footprint analysis (Supplemental Figure 18) also indicated a decrease in stride length (Figure 7H) and print length (Figure 7I) and an increase in sway length (Figure 7J) and toe spread (Figure 7K) in EAE mice as compared with normal mice. We also frequently observed dragging of toes in EAE mice (Supplemental Figure 18). However, intranasal treatment with WT TIDM, but not mTIDM, peptide improved locomotor activity and normalized the footprints of EAE mice (Figure 7, A–K, and Supplemental Figure 18). Collagen-induced arthritis (CIA) is a widely used animal model of rheumatoid arthritis. In EAE mice, WT TIDM, but not mTIDM, peptide also decreased clinical symptoms of CIA in (Figure 7L). When we compared the means between groups with Dunnett’s multiple comparisons analyses, we found that there was a significant difference of means between the CIA and CIA plus WT TIDM mice (adjusted *P* = 0.0148 [< 0.05]). WT TIDM peptide also enhanced locomotor activity (Figure 7, N–R) and improved footprint behavior in the mice (Figure 7, S–V, and Supplemental Figure 19).

## Discussion

Deciphering the mechanism of the disease process of AD and developing an effective neuroprotective therapeutic approach to slow down or halt the disease progression are of paramount importance. TLRs are known to resolve innate immune responses by perceiving pathogen-associated molecular patterns and endogenous damage-associated molecular patterns (15). Microglia in the CNS express most of the TLRs known to date, and earlier we showed that among the different TLRs, fibrillar Aβ1-42 requires TLR2 to stimulate microglial inflammation (17) . Accordingly, several studies have extended this finding either by demonstrating a direct interaction between TLR2 and Aβ or via CD14 (18, 19, 33). Here, we describe an important role of TLR2 in AD. We detected higher levels of TLR2 in the hippocampus and PFC of individuals with AD dementia versus those with MCI or NCI. Although some studies reported the involvement of TLR4 in Aβ-mediated microglial activation, we did not find higher levels of TLR4 in the CNS of individuals with AD dementia, indicating the specificity of our finding. *Tlr2* polymorphism has been reported to influence susceptibility to AD (34), and peripheral blood mononuclear cells (PBMCs) from patients with AD also express increased levels of TLR2 (35). Consistent with what was observed withTLR2, we also detected the upregulation of MyD88 in the CNS of individuals with AD dementia, and, interestingly, both TLR2 and MyD88 were positively correlated with the Braak score. MyD88 was also negatively correlated with cognitive function.

Although TLR2 is an important member of the innate immune system, we found no specific inhibitor targeting TLR2. Therefore, through structural analysis of the interaction between TLR2 and MyD88, we designed a peptide corresponding to the TIDM from the CD loop. Since the BB loop of TLR2 interacts with the CD loop of MyD88, WT TIDM peptide disrupts the association between TLR2 and MyD88. Interestingly, the WT TIDM peptide docks in a way that allows it to specifically target the BB loop of TLR2, but not other TLRs, thereby inhibiting signaling pathways transduced by TLR2 only. Since the WT TIDM peptide specifically targets TLR2 and fibrillar Aβ1-42 requires TLR2 for microglial activation (17, 18), WT TIDM peptide inhibited the microglial NF-κB activation and inflammation induced only by LTA (a known agonist of TLR2) and fibrillar Aβ1-42, but not by MPP^+^, poly IC (an agonist of TLR3), LPS (an agonist of TLR4), flagellin (an agonist of TLR5), or CpG DNA (an agonist of TLR9), indicating selective inhibition of the TLR2 pathway by WT TIDM peptide. Moreover, consistent with the disruption of TLR2-MyD88 interaction, WT TIDM peptide did not function in the absence of TLR2.

Unmodified peptides usually have short half-lives due to rapid proteolysis in blood, kidneys, or liver, and/or accelerated renal clearance, which are the major challenges of most peptide therapies. However, it has been shown that the *Drosophila* *antennapedia* homeodomain–derived cell-penetrating peptide penetratin, being rich in positively charged residues, helps cargo peptides translocate into the cells, therefore avoiding rapid proteolysis (36, 37). Moreover, unmodified peptides do not enter into the CNS, and we have seen that penetratin can breach the tight endothelial network and carry peptides across the blood-brain barrier (BBB) (23, 38). Therefore, we tested the efficacy of penetratin-containing WT TIDM peptide in Tg mice and demonstrated that WT TIDM peptide reduced microglial inflammation, decreased neuronal apoptosis, and protected cognitive function from AD toxicity. Our conclusions are based on several observations. First, after intranasal administration, TIDM peptide entered into the hippocampus. Second, WT TIDM, but not mTIDM, peptide inhibited hippocampal activation of NF-κB and microglial inflammation in Tg mice. Third, WT TIDM, but not mTIDM, peptide protected hippocampal neurons and NMDA and AMPA receptor proteins from AD toxicity in Tg mice. Fourth, WT TIDM, but not mTIDM, peptide also improved spatial learning and memory in Tg mice. Furthermore, we detected no drug-related side effects (e.g., hair loss, appetite loss, weight loss, untoward infection, etc.) in any of the TIDM-treated mice used during the course of the study. However, 1 study has shown that genetic knockdown of TLR2 accelerates the cognitive decline in APP-Tg mice (39). This is definitely possible, as complete knockdown of TLR2 wipes out basal as well as induced TLR2 signaling pathways. Moreover, TLR2 has been shown to function via both MyD88-dependent and -independent pathways (40, 41), and the beauty of our finding is that the TIDM peptide targeted only the MyD88-dependent induced TLR2 signaling pathway, without inhibiting basal TLR2 activity.

Whether or not plaques are directly related to the loss of memory in AD, amyloid plaque is one of the pathological hallmarks of this disease. It is also important to note that WT TIDM, but not mTIDM, peptide treatment reduced hippocampal plaque load in Tg mice. However, at present, we do not know how WT TIDM peptide treatment is coupled to plaque reduction. β-Secretase 1 (BACE1) is the key enzyme that initiates the formation of Aβ, and it has been shown that inhibition of NF-κB prevents Aβ-induced BACE1 promoter transactivation and that overexpression of WT or Swedish mutated βAPP does not modify the transactivation of BACE1 promoter constructs lacking the NF-κB–responsive element (42). Since WT TIDM peptide suppresses fibrillar Aβ–induced activation of NF-κB, it is possible that WT TIDM peptide reduces the plaque burden in Tg mice via attenuation of the NF-κB/BACE1 pathway.

There is no effective therapy for halting the progression of AD. Administration of different inhibitors of cholinesterase such as Aricept, Exelon, Razadyne, Cognex, etc., has been the standard treatment for this disease (43). However, this treatment is often associated with a number of side effects and unsatisfactory outcomes. Here, we have demonstrated that TLR2 and MyD88 levels are upregulated in the CNS of patients with AD; that TLR2 and MyD88 are positively correlated with the Braak score, that WT TIDM peptide targets only TLR2 without modulating other signaling pathways; and that after intranasal administration, WT TIDM peptide reaches the hippocampus, suppresses hippocampal NF-κB activation, inhibits microglial inflammation, lowers cerebral plaque load, attenuates neuronal apoptosis, and protects learning and memory functions in Tg mice. These results suggest that selective targeting of TLR2 by intranasal WT TIDM peptide may have therapeutic importance in AD. Moreover, WT TIDM peptide also improved functional impairment and suppressed disease processes of EAE and CIA in mice. Therefore, in addition to AD, TIDM peptide may also open up an opportunity for the treatment of other disorders.

## Methods

### Human subjects.

Samples from 33 subjects with an antemortem clinical diagnosis of NCI (*n* = 12), MCI (*n* = 11), or AD (*n* = 10) obtained from the Rush Religious Order Study (RROS) (44, 45) were analyzed (Supplemental Table 1). All participants agreed to a detailed annual clinical evaluation and brain donation upon their death.

### Clinical and neuropathologic evaluations.

The clinical criteria for the diagnosis of NCI, MCI, and AD have been reported elsewhere (44, 46). Final clinical and neuropsychological testing, which included the MMSE and a battery of 19 cognitive tests, was performed within 2 years before the participant’s death. A GCS comprising the 19 tests was available for all cases (47). Braak staging of NFTs (48) was performed as previously described (44). Subjects with pathological findings other than AD (e.g., stroke, Parkinson disease, Lewy body dementia) were excluded from the study.

### Tissue samples and Western blot analysis.

Superior frontal cortex (Brodmann area 9) was dissected free of white matter at autopsy on dry ice to prevent thawing and was maintained at −80°C until assay. Tissue was homogenized and processed as described previously (22). Tissue extracts and cell lysates (30 μg) were electrophoresed on 8% or 10% Bis-Tris SDS polyacrylamide gels in a continuous buffer system, transferred onto nitrocellulose membranes (Bio-Rad) with a semi-dry blotter (Pierce, Thermo Fisher Scientific), and immunoblotted as described previously (22, 49–51). Blots were converted to binary, analyzed using ImageJ (NIH), and normalized to the β-actin loading control.

### Preparation of cTLR2.

The TLR2 full-length construct (*pLenti-cMyc-DDK/Tlr2)* was purchased from Origene. cTLR2 (640–784 amino acids) tagged with cMyc was subcloned into a lentivector using the TOPO TA Cloning Kit (K5310-00; Life technologies, Thermo Fisher Scientific). Briefly, a Kozak sequence was incorporated upstream of the cTIR2 domain of TLR2. Next, cTLR2 was subcloned into the lentivector, followed by packaging in lentivirus using HEK293FT cells. After 48 hours, media were collected and concentrated with Lenti-X Concentrator (catalog 631231; Clontech). This concentrated lentiviral supernatant was used for viral transduction. The cTLR2 protein was isolated from the HEK293 cell lysate by passing through a Myc affinity column. Purified protein was desalted and concentrated by using a 10-kDa molecular cutoff filtration system.

### SPR.

To analyze the binding of TLR2 with TIDM peptides, SPR experiments were carried out using a Reichert 4SPR instrument. The binding assay was performed using a 500-kDa Carboxymethyl Dextran Gold Sensor Slide (Reichert Technologies) to capture TLR2. Protein immobilization was at a flow rate of 30 μl/min in PBS for 3 minutes with a 0.8-mg/ml solution of TLR2. For analyte association, different concentrations of WT TIDM and mTIDM peptides in PBS running buffer were injected for 2.5 minutes at a rate of 30 μl/min, followed by a dissociation phase of 3 minutes. The sensor surface was regenerated after each dissociation cycle by allowing buffer to flow at 40 μl/min for a minimum of 15 minutes. Signals obtained for the TLR2-bound surface were subtracted by signals obtained for the reference cell according to standard procedures using Reichert system software. The concentration dependence of the subtracted signal was analyzed to determine binding affinity of TLR2 with WT TIDM and mTIDM peptides.

### Thermal shift assays.

Thermal shift assays were performed on an Applied Biosystems 7500 standard real-time thermal cycler machine as described previously (52, 53). For each reaction, purified protein (0.5–1 μg) was added to 18 μl of the thermal shift buffer provided with the kit and 1–2 μl dye. The reaction was performed in a 96-well PCR plate in the dark and then placed in the thermal cycler machine using the following 2-stage program: 1 cycle at 25°C for 2 minutes; 70 cycles at 27°C for 15 seconds and 26°C for 1 minute; and auto increment at 1°C for both stages. The filter was set at ROX with no quencher filter and no passive filter.

### In silico structural analysis.

We used Deep View 3.7β2, an online macromolecular analytical tool of Expert Protein Analytical System (ExPASy), to model structures of TIR domains of different TLRs (TLR1, TLR2, TLR4, TLR5, TLR6, TLR7, and TLR9). In order to evaluate the quality of modeled structures, we used the Quality Measurement Analysis (QMEAN) tool, a composite scoring tool that estimates the global quality of the entire model as well as the local per-residue value of different regions within a model. Residue-level interaction was evaluated by the Cβ atom potential, and long-range interactions were validated by the all-atom potential. A solvation potential was implemented to analyze the burial status of the residues. The local geometry of each structure was analyzed by a torsion angle potential over 3 consecutive amino acids. The docked pose of the TIR domains with either the WT TIDM or mTIDM peptide was derived from the pyDock rigid-body, protein-protein docking tool (https://life.bsc.es/pid/pydockweb/).

### Animals and intranasal delivery of TIDM peptides.

B6SJL-Tg(APPSwFlLon,PSEN1*M146L*L286V)6799Vas/J–Tg (5XFAD, referred to here as Tg) mice were purchased from The Jackson Laboratory. Six-month-old male Tg mice were treated intranasally with WT TIDM or mTIDM peptides (0.1 mg/kg BW/2 d) for 30 days. Briefly, TIDM peptides were dissolved in 5 μl normal saline, mice were held in supine position, and saline was delivered into 1 nostril using a pipetman.

### Induction of chronic EAE and treatment with TIDM peptides.

Male C57BL/6 mice were immunized with 100 μg MOG35-55 as described by us previously (54, 55). Mice also received 2 doses of pertussis toxin (150 ng/mouse) 0 and 2 days post immunization (dpi). Starting from 10 dpi, mice received WT TIDM or mTIDM peptides (0.1 mg/kg BW/d) intranasally.

### Induction of CIA and treatment with TIDM peptides.

Male DBA/1J mice (8–9 weeks of age) were immunized intradermally at the base of the tail with 100 μg bovine type II collagen emulsified in incomplete Freund’s adjuvant and *M*. *tuberculosis* H37RA. On day 21 after immunization, the mice were boosted with an intraperitoneal injection of 100 μg bovine type II collagen. Mice were treated intraperitoneally with WT TIDM or mTIDM peptides (1 mg/kg BW/d) starting from 29 dpi.

### Preparation of fibrillar Aβ1-42.

Fibrillar Aβ1-42 (AnaSpec) was prepared by incubating freshly solubilized peptides at 50 μM in sterile distilled water at 37°C for 5 days (56). Supplemental Figure 20 shows the morphology of fibrillar Aβ1-42.

### Semiquantitative reverse transcriptase–coupled PCR analysis.

Total RNA was isolated from hippocampus using Ultraspec II RNA Reagent (Biotecx Laboratories Inc.) according to the manufacturer’s protocol. To remove any contaminating genomic DNA, total RNA was digested with DNase. Semiquantitative reverse transcriptase–coupled PCR was performed as described earlier (23, 57) using a RT-PCR Kit (Clontech).

### Real-time PCR analysis.

DNase-digested RNA was analyzed by real-time PCR using the ABI-Prism7700 Sequence Detection System (Applied Biosystems) as described previously (23, 57).

### EMSA.

Nuclear extracts were isolated, and EMSA was carried out as described before (22, 23).

### Barnes maze and T-maze tests.

Maze experiments were performed as described previously by us (52, 57). Briefly, for the Barnes maze test, mice were trained for 2 consecutive days, followed by testing on day 3. After each training session, the maze and escape tunnel were thoroughly cleaned with a mild detergent so the mice would not instinctively avoid odors on familiar objects in the maze. On day 3, the maze was illuminated with high-wattage light that generated enough light and heat to motivate animals to enter into the escape tunnel, allowing us to measure latency (duration before all 4 paws were on the floor of the escape box) and errors (incorrect responses before all 4 paws were on the floor of the escape box).

For the T-maze test, mice were also habituated in the T-maze for 2 days under food-deprived conditions so that the animals would eat food rewards at least 5 times over a 10-minute period of training. During each trial, mice were placed at the starting point for 30 seconds and then forced to turn at the right arm of the maze, which was always baited with colored food chips. After each training session, the T-maze was thoroughly cleaned with a mild detergent. On day 3, mice were tested for making positive turns and negative turns. The reward side was always associated with a visual cue. Each time the animal ate the food reward, it was considered a positive turn.

### NOR task.

The NOR task was performed to monitor short-term memory as described by others (58) and us (57). Briefly, during training, the mice were placed in a square novel box (20 in. long × 8 in. high) surrounded with an infrared sensor. Two plastic toys (2.5–3 in. size) that varied in color, shape, and texture were placed in specific locations in the environment 18 inches away from each other. The mice were able to freely explore the environment and objects for 15 minutes and were then placed back into their individual home cages. After 30 minutes, the mice were placed back into the environment, with the 2 objects in the same locations, but now 1 of the familiar objects was replaced with a third novel object. The mice were again allowed to freely explore both objects for 15 minutes. The objects were thoroughly cleaned with a mild detergent after each session.

### IHC.

Mice were anesthetized with ketamine-xylazine injectables and perfused with PBS and then with 4% (w/v) paraformaldehyde in PBS, followed by dissection of the brain for immunofluorescence microscopic examination (23, 59). Briefly, samples were incubated in PBS containing 0.05% Tween 20 (PBST) and 10% sucrose for 3 hours and then 30% sucrose overnight at 4°C. Brain tissue was then embedded in OCT (Tissue Tech) at –80°C and processed for conventional cryosectioning. Frozen sections (30-μm-thick) were treated with cold ethanol (–20 °C), followed by 2 rinses in PBS, blocking with 3% BSA in PBST, and double labeling with 2 antibodies (Supplemental Table 3). After 3 washes in PBST, the sections were further incubated with Cy2 and Cy5 (Jackson ImmunoResearch Laboratories). The samples were mounted and observed under an Olympus IX81 fluorescence microscope. Counting analysis was performed using Olympus Microsuite V software with the help of a touch counting module.

### Fragment end-labeling of DNA.

Fragment end-labeling of DNA was performed using a commercially available kit (TdT FragEL, Calbiochem) as described previously (10, 22).

### ELISA for Aβ_1-42_ and Aβ_1-40_.

Hippocampal tissues were homogenized in TBS and pelleted for 30 minutes at 150,000 *g*. The pellet was resuspended in 3 volumes (w/v original tissue weight) of TBS plus 1% Triton X-100, pelleted for 30 minutes at 150,000 *g*, and the supernatant recovered and stored. Samples were assayed for protein concentration and diluted 10-fold prior to performing ELISA according to the manufacturer’s instructions (BioLegend).

### Statistics.

Clinical and biochemical data on human tissues were compared across diagnoses using nonparametric tests (i.e., Kruskal-Wallis test or Fisher’s exact test, with Dunn’s correction for multiple comparisons), which are more robust to outliers, non-normality, and unequal sample sizes. A 2-tailed Spearman’s rank-order correlation was used to assess variable associations between cognitive test scores and protein optical densities. Correlations were unadjusted for demographic information (i.e., age, sex, etc.), as these metrics were not significantly different between clinical groups. Statistical tests were performed using SPSS 19 (IBM), and significance was set at *P* = 0.05 (2-sided). Mouse behavioral measures were calculated by an independent 1-way ANOVA using SPSS. Homogeneity of variance between test groups was examined using Levene’s test. Post-hoc analyses were conducted using Tukey’s or Games-Howell tests, where appropriate. Other data are expressed as the mean ± SD of 3 independent experiments. Statistical differences between means were calculated by 2-tailed Student’s *t* test. A *P* value of less than 0.05 was considered statistically significant.

### Study approval.

The Human Investigations Committee of Rush University Medical Center approved the RROS. Tissue and clinical information is under the protection of Health Information Privacy Administration rules. Animal care and experiments were conducted in accordance with NIH guidelines and approved by the IACUC of Rush University Medical Center.

## Author contributions

SBR, AR, MJ, and GTC designed and performed experiments. MK, SC, SM, SD, RKM, and CHL performed experiments. EJM and DAB provided reagents and wrote the manuscript. KP designed the project and wrote the manuscript.

## Supplementary Material

Supplemental data

## Version 1 07/10/2018

In-Press Preview

## Version 2 09/04/2018

Electronic publication

## Version 3 10/01/2018

Print issue publication
