# Supplementary material for: Selective disruption of TLR2-MyD88 interaction inhibits inflammation and attenuates Alzheimer’s pathology
Source: J Clin Invest. 2018 Jul 10;128(10):4297–312. doi: 10.1172/JCI96209 (PMC6159992; doi:10.1172/JCI96209)
Supplement: Supplemental data [file jci-128-96209-s177.pdf]

Selective disruption of TLR2/MyD88 interaction inhibits inflammation and attenuates Alzheimer's pathology

Suresh B. Rangasamy[1,*], Malabendu Jana[1,*], Avik Roy[1,*], Grant T. Corbett[1,*,&], Madhuchhanda Kundu[1], Sujyoti Chandra[1], Susanta Mondal[1], Sridevi Dasarathi[1], Elliott J. Mufson[2], Rama K. Mishra[3], Chi-Hao Luan[4], David A. Bennett[5], and Kalipada Pahan[1,6]

[1]Department of Neurological Sciences, Rush University Medical Center, Chicago, IL; [2]Barrow Neurological Institute, Phoenix, AZ; [3]Medicinal and Synthetic Chemistry Core, Center for Molecular Innovation and Drug Discovery, Northwestern University, Evanston, IL; [4]High Throughput Analysis Laboratory and Department of Molecular Biosciences, Northwestern University, Evanston, IL; [5]Rush Alzheimer's Disease Center, Rush University Medical Center, Chicago, IL; [6]Division of Research and Development, Jesse Brown Veterans Affairs Medical Center, 820 South Damen Avenue, Chicago, IL

[*]First four authors have equal contribution to the work.

[&]Current address: Ann Romney Center for Neurologic Diseases, Brigham and Women's Hospital, Harvard Medical School, Boston, MA

Conflict of interest: None

Running title: Selective inhibition of TLR2 activation by TIDM peptide

Corresponding author with complete address:
Kalipada Pahan, Ph.D.
Department of Neurological Sciences
Rush University Medical Center
1735 West Harrison St, Suite 310
Chicago, IL 60612
Tel: (312) 563-3592
Fax: (312) 563-3571
Email: Kalipada_Pahan@rush.edu

Legends to Supplemental Figures

Figure S1. Monitoring TLR2, TLR4 and MyD88 in the CNS of cases clinically diagnosed as no cognitive impairment (NCI) and Alzheimer's disease (AD). Hippocampal sections of NCI and AD brains were double-labeled with Iba-1 (microglia) & TLR2 (A), Iba-1 & MyD88 (B) and Iba-1 & TLR4 (C). Results represent analysis of two sections from each of four different brains. Cells positive for Iba1 (D, cortex; E, CA1) were counted in two sections (two images per slide) of each of four different cases. $^{a}p < 0.001$ *vs NCI* by two-sample t-tests.

Figure S2. Status of TLR2 in the CNS of non-Tg and Tg (5XFAD) mice. A) Hippocampal sections of six-month old non-Tg and Tg mouse brains were double-labeled with Iba-1 (microglia) and TLR2. Results represent analysis of two sections from each of six different mice. B) Hippocampal extracts of all groups of mice (n=4) were immunoblotted for TLR2. Actin was run as loading control. C) Bands were scanned using the NIH Image J software and values (TLR2/Actin) are presented as relative to non-Tg control. $^{a}p < 0.001$ *vs non-Tg* by two-sample t-tests.

Figure S3. Status of MyD88 in the CNS of non-Tg and Tg (5XFAD) mice. A) Hippocampal sections of six-month old non-Tg and Tg mouse brains were double-labeled with Iba-1 (microglia) and MyD88. Results represent analysis of two sections from each of six different mice. B) Hippocampal extracts of all groups of mice (n=4) were immunoblotted for MyD88. Actin was run as loading control. C) Bands were scanned using the NIH Image J software and values (MyD88/Actin) are presented as relative to non-Tg control. $^{a}p < 0.001$ *vs non-Tg* by two-sample t-tests.

Figure S4. Implementation of in silico homology modeling strategy to build the structure of TLR-interacting domain of different mouse TLRs. Initial structures of TIRs (A, TLR1; B, TLR2; C, TLR4; D, TLR5; E, TLR6; F, TLR7; G, TLR9) were modeled by Deep View 3.7β2, an online macromolecular analytical tool of Expert Protein Analytical System (ExPASy). The quality of each modeled structure was evaluated with Quality Measurement Analysys tool (QMEAN).

Figure S5. Docking analyses of wtTIDM and mTIDM complexed with TIR domain of TLR2 protein. A) *In silico* structural analysis of TIR domain of TLR2 and wtTIDM peptide. The docked pose was derived from pydock rigid-body protein-protein docking tool. The most stable structure was obtained from j mol viewer and displayed. B) Similar analysis was performed with mTIDM. C) Further analyses revealed a strong electrostatic interaction between wtTIDM and TLR2 (~2.31 A°: *left*) and a weak interaction with mTIDM (~7.26A°; *right*) (D) A closer look of a complex between BB loop of TLR2 (blue) and CD loop of MYD88 (green). VDW droplets were shown to be overlapped with each other. E) The VDW clouds of MYD88 (pink) moved far from these of TLR2 (blue) when complexed with wtTIDM (green).

Figure S6. Effect of wtTIDM and mTIDM peptides on the expression of proinflammatory molecules in microglial cells. BV-2 microglial cells preincubated with 10 μM wtTIDM/mTIDM peptides for 1 h were stimulated with 1 μM fibrillar Aβ1-42 (A), 1 μM MPP^{+} (B), 250 ng/ml LTA (C), 1 μg/ml LPS (D), 1 μM flagellin (E), and 1 μM CpG DNA (F). After 4 h of stimulation, the mRNA expression of IL-1β and iNOS was monitored by RT-PCR (n=2 replicates/condition in 3 independent experiments).

Figure S7. Effect of wtTIDM and mTIDM peptides on polyIC-mediated activation of NF-κB activation and the expression of proinflammatory molecules in microglial cells. BV-2 microglial cells preincubated with 10 μM wtTIDM/mTIDM peptides for 1 h were stimulated with 50 μM polyIC. A) After 1 h of stimulation, the activation of NF-κB was monitored in nuclear extracts by EMSA. After 4 h of stimulation, the mRNA expression of IL-1β and iNOS was monitored by semi-quantitative RT-PCR (B) and real-time PCR (C, IL-1β; D, iNOS) (n=2 replicates/condition in 3 independent experiments). $^{a}p < 0.001$ *vs control;* $^{b}p < 0.001$ *vs stimuli* by two-sample t-tests.

Figure S8. Effect of wtTIDM peptide on fibrillar Aβ- and LPS-induced nuclear translocation of p65 and p50 in microglial cells. BV-2 microglial cells preincubated with 10 μM wtTIDM peptide for 1 h were stimulated with either 1 μM fibrillar Aβ1-42 (A-C) or 1 μg/ml LPS (D-F) under serum-free condition. At different minute intervals, levels of p65 and p50 (A, fibrillar Aβ; D, LPS) were monitored in nuclear extracts by Western blot. Histone H3 was run as a loading control. Bands were scanned and values of p65/H3 (B & E) and p50/H3 (C & F) are presented as relative to control (n=2 replicates/condition in 3 independent experiments). $^{a}p < 0.05$, $^{b}p < 0.001$ *vs control;* $^{c}p < 0.01$ *vs 30 min stimulation;* $^{d}p < 0.05$ *vs 15 min stimulation; NS, not significant* by two-sample t-tests.

Figure S9. The wtTIDM peptide remained unable to inhibit fibrillar Aβ1-42 peptide-mediated activation of NF-κB and the expression of proinflammatory molecules in TLR2 (-/-) microglia. Primary microglia isolated from WT (A) and TLR2 (-/-) (B) mice were treated with different concentrations of wtTIDM peptide for 1 h followed by stimulation with 1 μM fibrillar Aβ1-42 under serum-free condition. After 1 h of stimulation, the activation of NF-κB was monitored by EMSA. WT (C & D) and TLR2 (-/-) (E & F) microglia were treated with different concentrations of wtTIDM and mTIDM peptides for 1 h followed by stimulation with 1 μM fibrillar Aβ1-42 under serum-free condition. After 18 h of stimulation, levels of TNFα (C & E) and IL-1β (D & F) were monitored in supernatants by ELISA (n=2 replicates/condition in 3 independent experiments). $^{a}p < 0.001$ *vs control;* $^{b}p < 0.001$ *vs stimuli* by two-sample t-tests.

Figure S10. After intranasal administration, wtTIDM peptide enters into the hippocampus of Tg mice. The wtTIDM peptide was labeled with Alexa 680-SE NIR dye (Life Technologies) following the manufacturer's protocol and Alexa 680-labeled peptide (2.5 μg) was administered to each mouse intranasally. Alexa 680-SE NIR dye was also administered as control. After 60 min, mice (n=3 in each group) were perfused with PBS and paraformaldehyde and hippocampal regions of the brain were scanned in Odyssey (ODY-0854, Licor-Inc) infra-red scanner at 700 and 800 nm channels. The red background came from 800 nm filter whereas the green signal was from Alexa 680-labeled NBD peptide at 700 nm channel.

Figure S11. After intranasal delivery, wtTIDM peptide suppresses the activation of NF-κB in the hippocampus of Tg mice. Tg mice (6-month old) were treated with wtTIDM and mTIDM peptides (0.1 mg/kg body wt/2d) via intranasal route. After 30d of treatment, hippocampal extracts of all groups of mice were immunoblotted for phospho-p65 (A). Actin was run as loading control. Bands were scanned and values (p-p65/Actin) are presented as relative to non-Tg control (n=4 in two independent experiments). $^{a}p < 0.001$ *vs non-Tg;* $^{b}p < 0.001$ *vs Tg* by two-sample t-tests.

Figure S12. Intranasal delivery of wtTIDM, but not mTIDM, peptide suppresses microglial expression of iNOS in the hippocampus of Tg mice. Tg mice (6-month old) were treated with wtTIDM and mTIDM peptides (0.1 mg/kg body wt/2d) via intranasal route. After 30d of treatment, mice were sacrificed and hippocampal sections were double-labeled for Iba-1 and iNOS. Results represent analysis of two sections from each of six different mice per group.

Figure S13. Intranasal delivery of wtTIDM, but not mTIDM, peptide lowers the burden of amyloid plaques in the hippocampus of Tg mice. A) Tg mice (6-month old) were treated with wtTIDM and mTIDM peptides (0.1 mg/kg body wt/2d) via intranasal route. After 30d of treatment, hippocampal extracts of all groups of mice (n=4 per group) were analyzed for protein levels of Aβ by Western blot using 82E1 mAb. Actin was run as loading control. B) Bands were scanned and values (Aβ/Actin) presented as relative to non-Tg control. Results were analyzed by two-sample t-tests.

Figure S14. Intranasal delivery of wtTIDM, but not mTIDM, peptide reduces the levels of $A\beta_{1-40}$ and $A\beta_{1-42}$ in serum and hippocampus of Tg mice. Tg mice (6-month old) were treated with wtTIDM and mTIDM peptides (0.1 mg/kg body wt/2d) via intranasal route. After 30d of treatment, ELISA quantification of $A\beta_{1-40}$ (A, C & E) and $A\beta_{1-42}$ (B, D & F) was performed in serum (A & B) and TBS (C & D) and (TBS+Triton X-100) (E & F) extracted hippocampal tissues. Six mice (n=6 per group) were used in two independent experiments. $^{a}p < 0.01$ & $^{c}p < 0.001$ versus *non-Tg*; $^{b}p < 0.01$ & $^{d}p < 0.001$ versus *Tg* by two-sample t-tests.

Figure S15. Intranasal delivery of wtTIDM, but not mTIDM, peptide decreases the phosphorylation of tau in the hippocampus of Tg mice. A) Tg mice (6-month old) were treated with wtTIDM and mTIDM peptides (0.1 mg/kg body wt/2d) via intranasal route. After 30d of treatment, hippocampal extracts of all groups of mice (n=4 per group) were analyzed for phospho-tau and total tau by Western blot. B) Bands were scanned and values (P-Tau/Tau) presented as relative to non-Tg control. Results were analyzed by two-sample t-tests.

Figure S16. Intranasal delivery of wtTIDM and mTIDM peptides does not modulate locomotor activities of Tg mice. Tg mice (6-month old) were treated with wtTIDM and mTIDM peptides (0.1 mg/kg body wt/2d) via intranasal route. After 30d of treatment, mice were tested for for general locomotor activities (A, number of movements; B, horizontal activity; C, rest time; D, number of stereotypy). Eight mice (n=8 in two independent experiments) were used in each group. NS, not significant.

Figure S17. Intranasal delivery of wtTIDM peptide does not reduce plaques and improve memory in FAD5X Tg mice lacking Tlr2 (Tg-*Tlr2*$^{-/-}$). A) *Tlr2*$^{-/-}$ mice were bred with Tg (5XFAD mice) and representative PCR of *Tlr2*, *App695 and Psen1* transgene DNA expression is shown for 6-month old non-Tg, Tg (5XFAD), Tg-*Tlr2*$^{-/-}$ (F7), and *Tlr2*$^{-/-}$ mice. Average body weight (B) and wet brain weight (C) of non-Tg, Tg, Tg-*Tlr2*$^{-/-}$, and *Tlr2*$^{-/-}$ mice. For wet brain weight, the olfactory lobes and brainstem were removed. Tg-*Tlr2*$^{-/-}$ mice (6-month old) were treated with wtTIDM peptide (0.1 mg/kg body wt/2d) via intranasal route. After 30d of treatment, hippocampal sections were immunolabeled with 6E10 mAb (D). Amyloid plaques were counted in two sections (two images per slide) of each of four different mice per group (E). Mice were tested for Barnes maze (F, track plot; G, latency; H, number of errors made). Four mice (n=4) were used in each group. NS, not significant by two-sample t-tests.

Figure S18. Footprint analysis of EAE mice after treatment with wtTIDM and mTIDM peptides. On the walking track, we applied white paper strips and obtained the footprints of mice of different groups (A, control; B, EAE; C, EAE+wtTIDM; D, EAE+mTIDM) on paper using black ink. A total of 30-40 steps for each group were determined. Four different footprint measurements, viz., stride length (SL), print length (PL), sway length (SWL), and toe spread (TS) were calculated in centimeters from the recorded prints of mice. While SL refers the distance between the front edge of two consecutive prints of the same paw, SWL refers the distance between the paws perpendicular to the distance of travel and PL indicates the measurement of length of print area. On the other hand, TS refers the distance between the first and fifth digits of paw print. Six mice (n=6 per group) were used in two independent experiments.

Figure S19. Footprint analysis of mice with CIA after treatment with wtTIDM and mTIDM peptides. On the walking track, we applied white paper strips and obtained the footprints of mice of different groups (A, control; B, CIA; C, CIA+wtTIDM; D, CIA+mTIDM) on paper using black ink. A total of 30-40 steps for each group were determined. Four different footprint measurements (SL, PL, SWL, and TS) were calculated in centimeters from the recorded prints of mice. Six mice (n=6 per group) were used in two independent experiments.

Figure S20. Morphology of fibrillar Aβ1-42 peptides. Fibrillar Aβ1–42 peptides (Bachem Bioscience) were prepared by incubating freshly solubilized peptides at 50 μM in sterile distilled water at 37 °C for 5 days. Morphology of fibrillar Aβ1–42 peptides was examined by transmission electron microscopy.

Table S1: Clinical and pathological characteristics of human samples

Number of samples	NCI (n=12)	MCI (n=11)	AD (n=10)
Age (years) at death	82.18±5.13	84.87±6.23	88.73±5.89
Number of males	4	7	5
Number of females	8	4	5
Number of ApoE e4 allele	4	2	5
MMSE	27.25±2.77	25.95±1.92	13.30±5.27
GCS	0.44±0.32	0.09±0.27	-1.13±0.39
PMI (hours)	7.45±6.36	5.15±3.12	6.57±3.33
Distribution of Braak Scores			
No AD	0	0	0
I/II	2	2	1
III/IV	10	7	5
V/VI	0	2	4
NIA Reagan			
No AD	0	0	0
Low	5	3	1
Intermediate	7	7	6
High	0	1	3
CERAD			
No AD	5	3	0
Possible	0	0	0
Probable	5	6	6
Definite	2	2	4

NCI, No cognitive impairment; MCI, mild cognitive impairment; AD, Alzheimer's disease; ApoE, apolipoprotein E; MMSE, Mini-Mental State Examination; GCS, global cognitive z score; PMI, post-mortem interval; NIA, National Institute on Aging; CERAD, Consortium to Establish a Registry for Alzheimer's Disease

Table S2: Correlations of TLR2, TLR4 and MyD88 with Cognitive Test Scores

	NCI	MCI	AD	p^a	Pairwise	r_s^b		
						MMSE	GCS	Braak
MyD88	6.38 ± 1.26	14.69 ± 4.41	45.66 ± 7.72	<.001	NCI, MCI < AD	-.538, *p* = .001	-.475, *p* = .005	.371, *p* = .033
TLR2	34.75 ± 6.05	29.98 ± 11.44	69.37 ± 11.47	.018	NCI, MCI < AD	-.278, *p* = .117	-.177, *p* = .326	.463, *p* = .007
TLR4	15.36 ± 3.82	14.61 ± 4.92	10.55 ± 1.61	.620	N/A	-.173, *p* = .336	.047, *p* = .794	-.012, *p* = .947

Pre-frontal cortex homogenates of NCI, MCI and AD were immunoblotted with antibodies against TLR2, TLR4 and MyD88. β-actin was used to normalize loading. Values represent mean ± SEM (range). Protein levels of TLR2, TLR4 and MyD88 were correlated with MMSE, GCS and Braak. AD, Alzheimer's disease; MCI, mild cognitive impairment; NCI, no cognitive impairment; MMSE, Mini-Mental State Examination; GCS, Global Cognitive *z* Score.
[a]Kruskal-Wallis test corrected for multiple comparisons; Spearman's Rank-Order correlation (2-tailed), unadjusted.

Table S3. **Antibodies, sources, applications, and dilutions used**

Antibody	Manufacturer	Catalog	Host	Application	Dilution/ Amount
TLR2	Millipore	06-1119	Rabbit	WB/IF	1:1000/1:100
TLR4	Abcam	Ab13556	Rabbit	WB/IF	1:1000/1:150
MyD88	Millipore	AB16527	Rabbit	WB	1:1000
MyD88	Abcam	Ab2068	Rabbit	IF	1:150
MyD88	Santa Cruz	Sc11356	Rabbit	IP	2 μg/reaction
β-actin	Abcam	Ab6276	Mouse	WB	1:6000
6E10	Covance	sig-39320	Mouse	WB	1:1000
phosphorylated $p65^{S536}$	Cell Signaling	3031S	Rabbit	WB/IF	1:1000
82E1	IBL	10323	Mouse	IHC	1:1000
Iba-1	Abcam	Ab5076	Goat	IF	1:500
GFAP	Dako	Z0334	Rabbit	WB/ IF	1:1000/1:2000
NeuN	Millipore	NAB377	Mouse	IF	1:500
iNOS	BD Bioscience	610432	Mouse	WB/IF	1:200
Cleaved caspase 3	Santa Cruz	sc-7148	Rabbit	WB	1:100
PSD95	Abcam	Ab2723	Mouse	WB	1:1000
NR2A	Cell Signaling	4205S	Rabbit	WB	1:250
GluR1	Cell Signaling	13185S	Rabbit	WB	1:250
P-Tau (pSer396)	Sigma-Aldrich	SAB4504557	Rabbit	WB/IF	1:1000
Tau (TAU-5)	Millipore-Sigma	577801	Mouse	WB/IF	1:1000

WB, Western blot; IP, immunoprecipitation; IHC, immunohistochemistry; IF, immunofluorescence

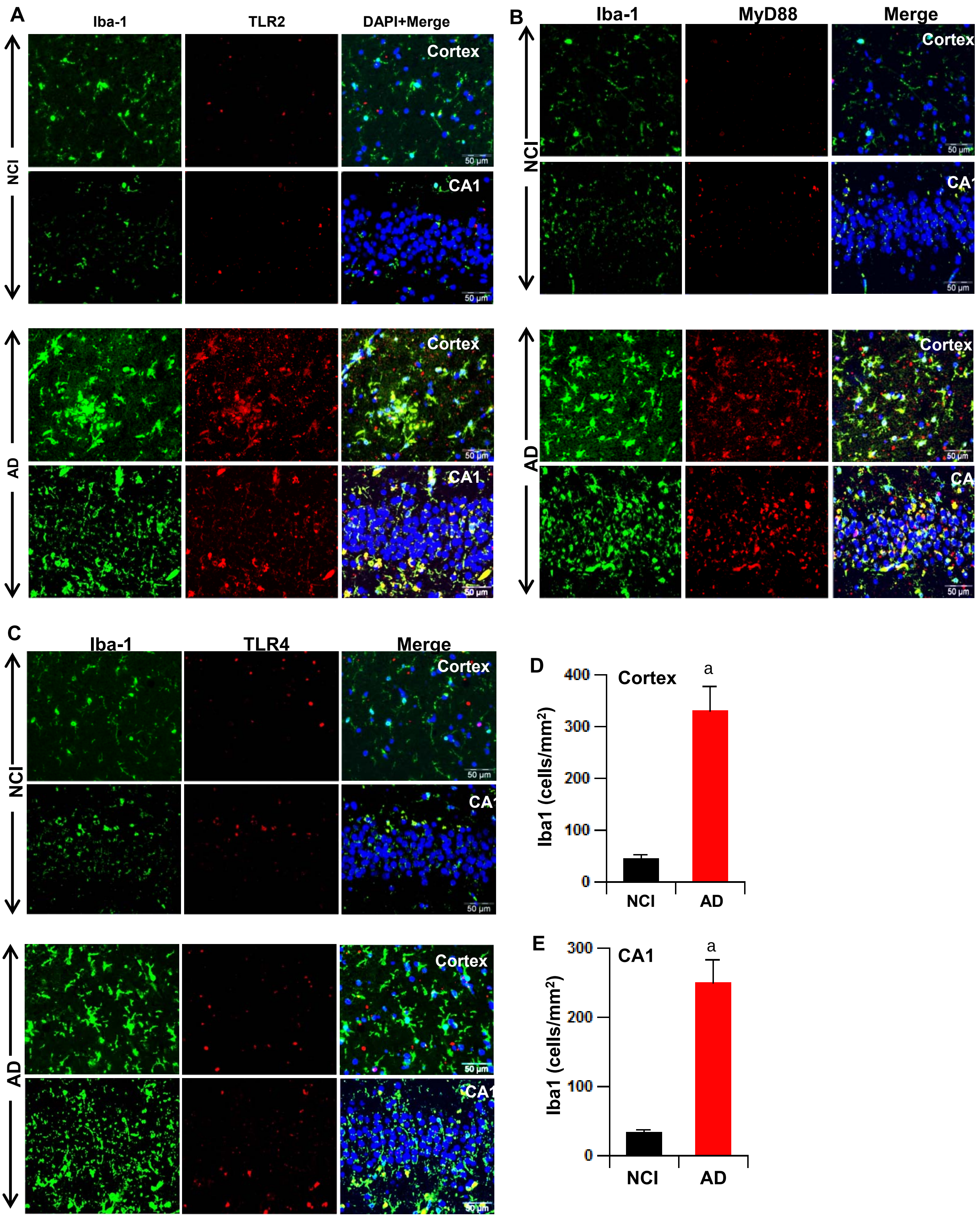

A
Iba-1
TLR2
DAPI+Merge
Cortex
CA1
NCI
AD
50 µm
B
Iba-1
MyD88
Merge
C
TLR4
D
Cortex
a
Iba1 (cells/mm²)
400
300
200
100
0
NCI
AD
E
CA1

Figure S1

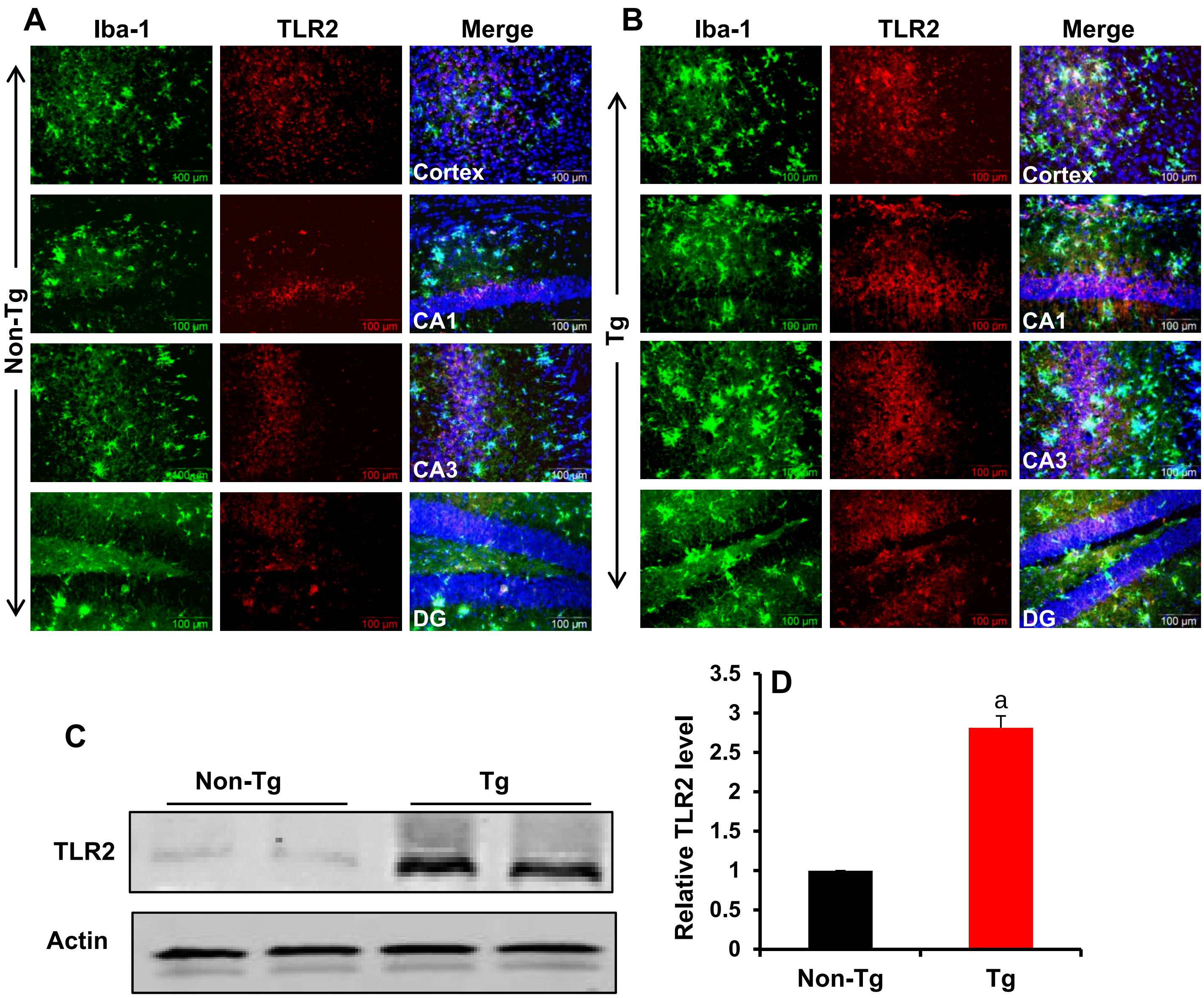
A
Iba-1
TLR2
Merge
Non-Tg
Cortex
CA1
CA3
DG
100 µm
B
Iba-1
TLR2
Merge
Tg
Cortex
CA1
CA3
DG
100 µm
C
Non-Tg
Tg
TLR2
Actin
D
Relative TLR2 level
3.5
3
2.5
2
1.5
1
0.5
0
a
Non-Tg
Tg

Figure S2

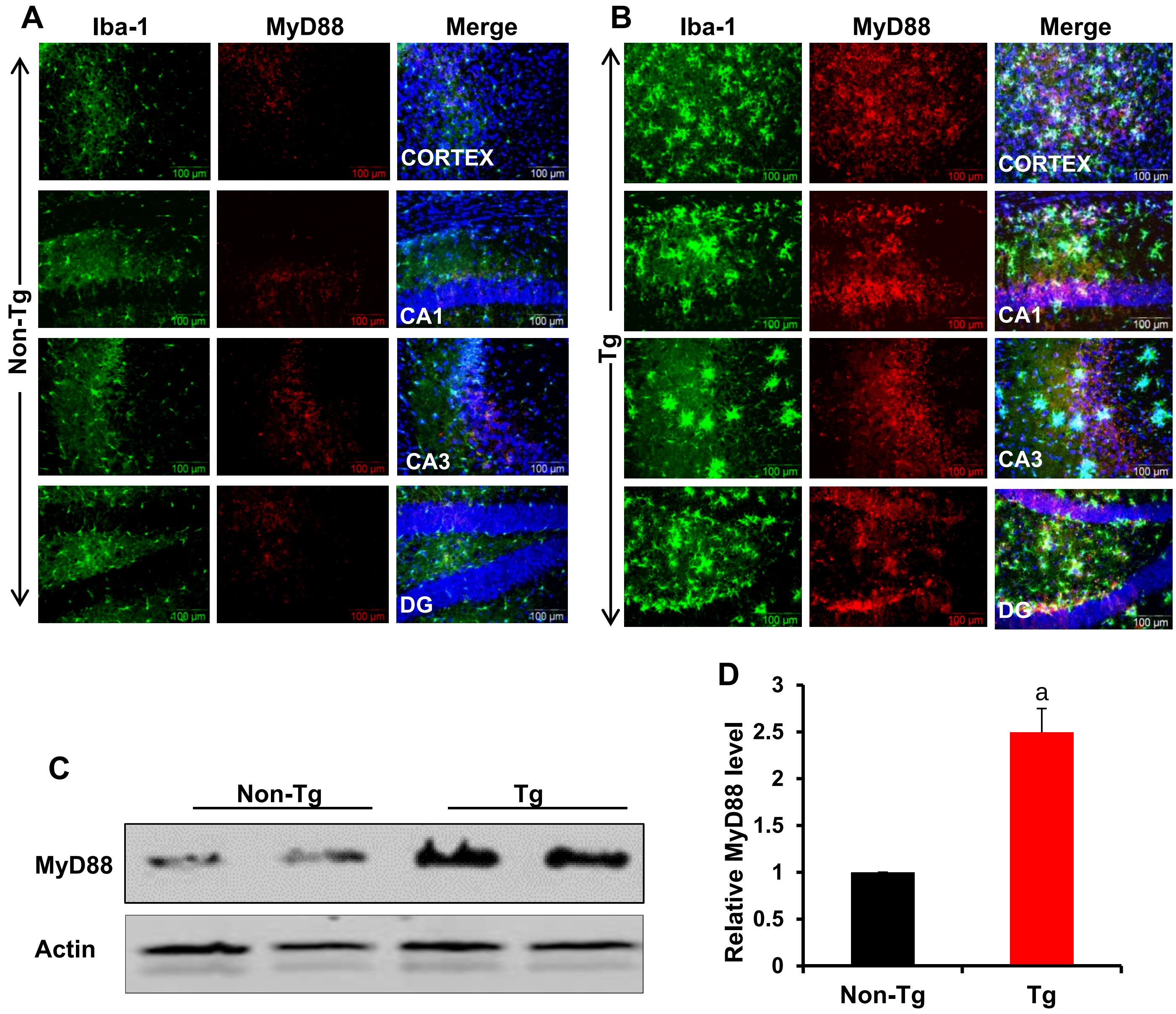
A
Iba-1
MyD88
Merge
Non-Tg
CORTEX
CA1
CA3
DG
100 µm
B
Iba-1
MyD88
Merge
Tg
CORTEX
CA1
CA3
DG
100 µm
C
Non-Tg
Tg
MyD88
Actin
D
Relative MyD88 level
3
2.5
2
1.5
1
0.5
0
a
Non-Tg
Tg

Figure S3

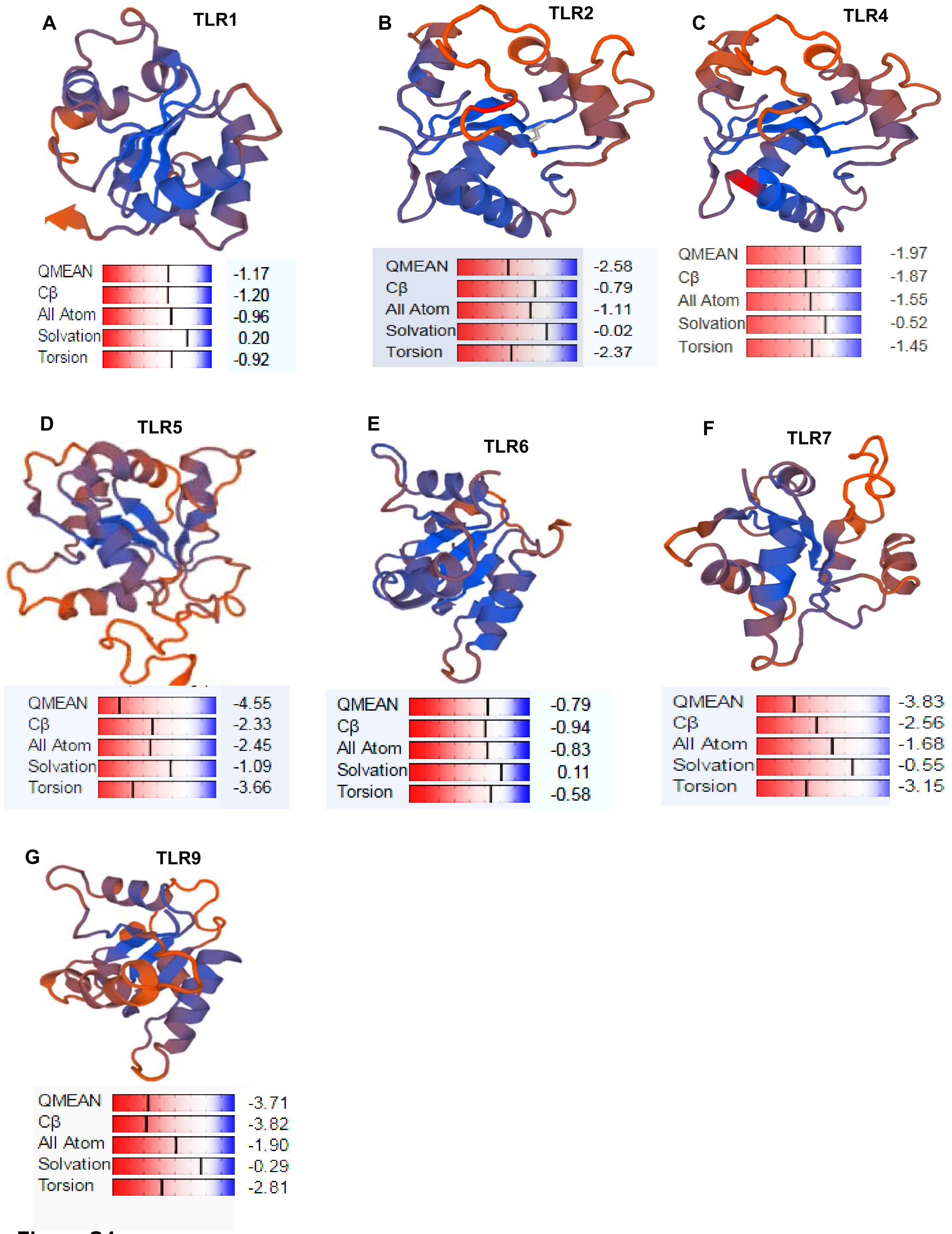
A
TLR1
QMEAN -1.17
Cβ -1.20
All Atom -0.96
Solvation 0.20
Torsion -0.92
B
TLR2
QMEAN -2.58
Cβ -0.79
All Atom -1.11
Solvation -0.02
Torsion -2.37
C
TLR4
QMEAN -1.97
Cβ -1.87
All Atom -1.55
Solvation -0.52
Torsion -1.45
D
TLR5
QMEAN -4.55
Cβ -2.33
All Atom -2.45
Solvation -1.09
Torsion -3.66
E
TLR6
QMEAN -0.79
Cβ -0.94
All Atom -0.83
Solvation 0.11
Torsion -0.58
F
TLR7
QMEAN -3.83
Cβ -2.56
All Atom -1.68
Solvation -0.55
Torsion -3.15
G
TLR9
QMEAN -3.71
Cβ -3.82
All Atom -1.90
Solvation -0.29
Torsion -2.81

Figure S4

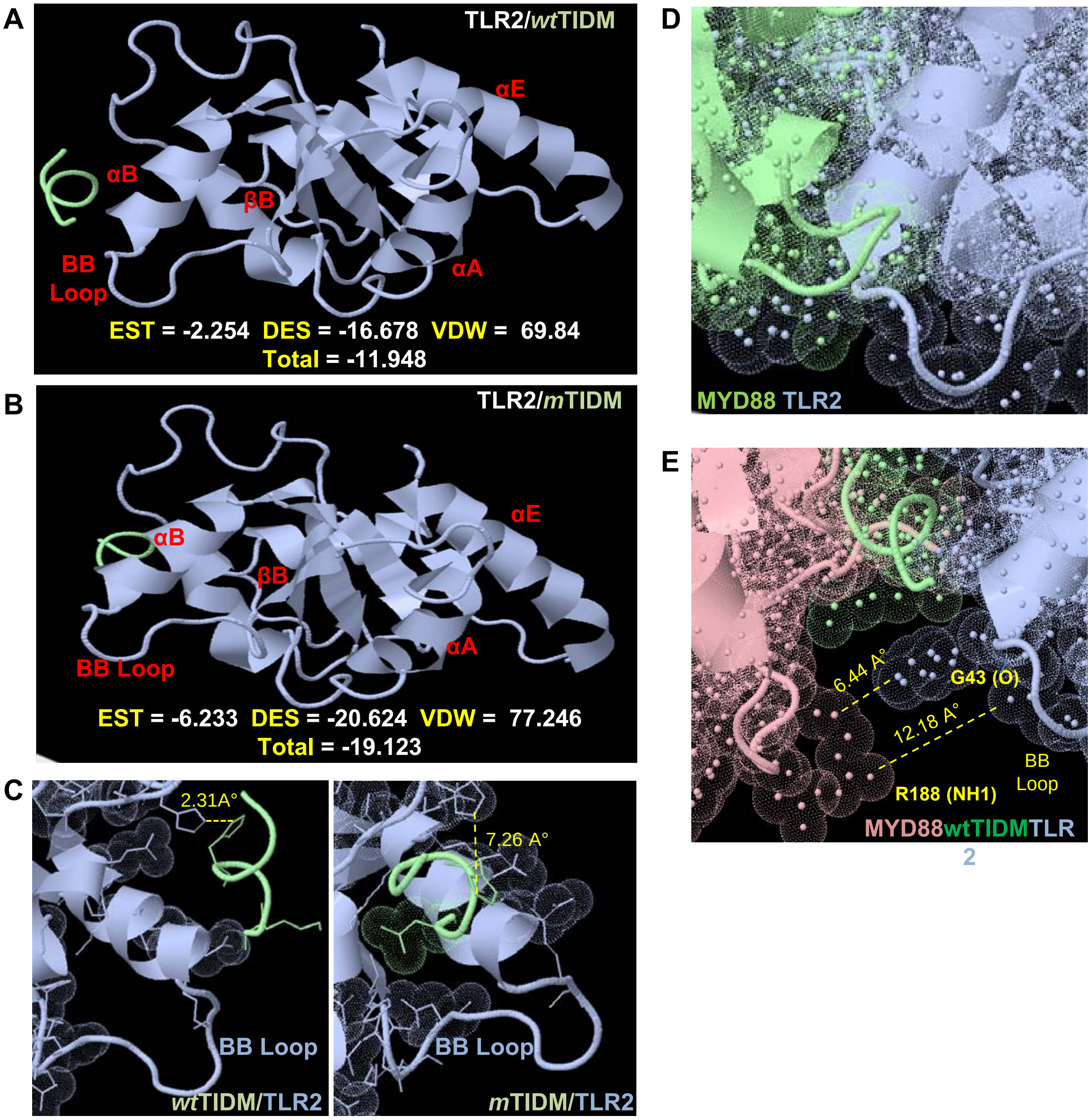
A
TLR2/wtTIDM
αE
αB
βB
BB
Loop
αA
EST = -2.254 DES = -16.678 VDW = 69.84
Total = -11.948
B
TLR2/mTIDM
αE
αB
βB
BB Loop
αA
EST = -6.233 DES = -20.624 VDW = 77.246
Total = -19.123
C
2.31A°
BB Loop
wtTIDM/TLR2
7.26 A°
BB Loop
mTIDM/TLR2
D
MYD88 TLR2
E
6.44 A°
G43 (O)
12.18 A°
BB
Loop
R188 (NH1)
MYD88wtTIDMTLR
2

Figure S5

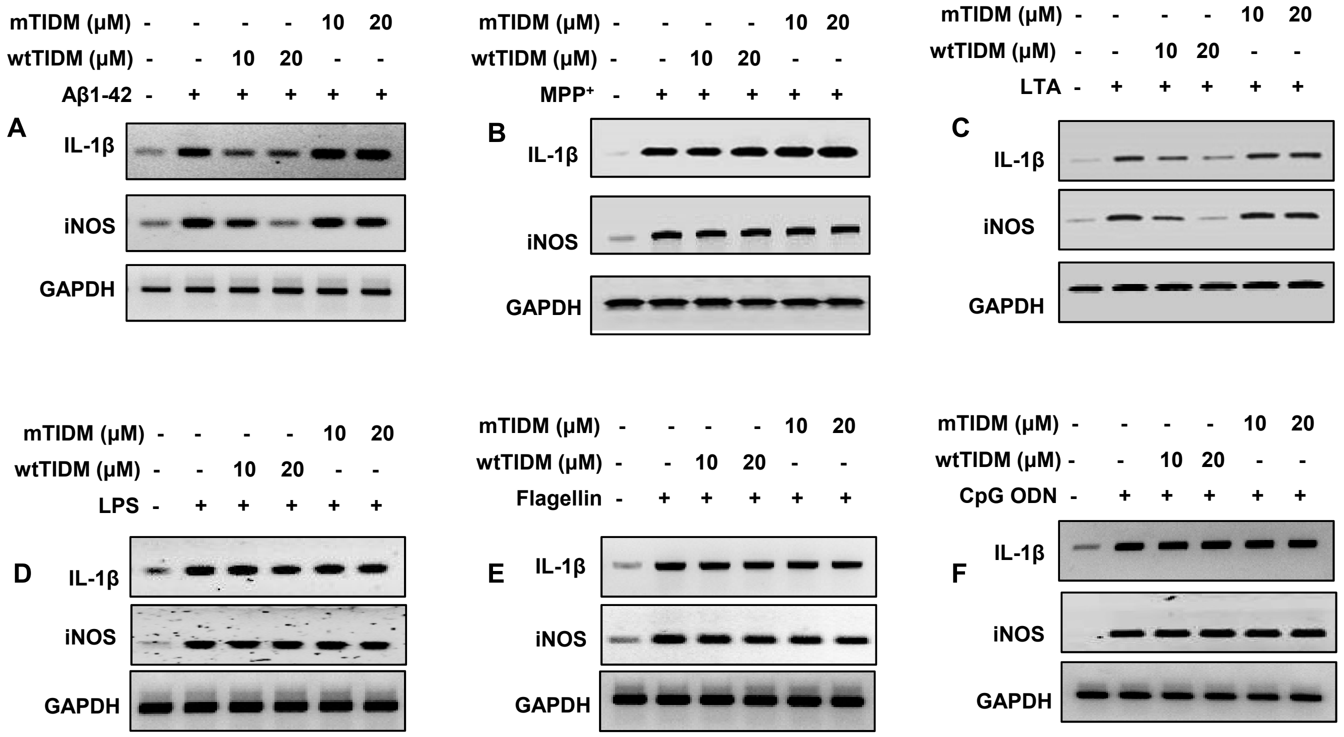
mTIDM (μM) - - - - 10 20
wtTIDM (μM) - - 10 20 - -
Aβ1-42 - + + + + +
A
IL-1β
iNOS
GAPDH
mTIDM (μM) - - - - 10 20
wtTIDM (μM) - - 10 20 - -
MPP+ - + + + + +
B
IL-1β
iNOS
GAPDH
mTIDM (μM) - - - - 10 20
wtTIDM (μM) - - 10 20 - -
LTA - + + + + +
C
IL-1β
iNOS
GAPDH
mTIDM (μM) - - - - 10 20
wtTIDM (μM) - - 10 20 - -
LPS - + + + + +
D
IL-1β
iNOS
GAPDH
mTIDM (μM) - - - - 10 20
wtTIDM (μM) - - 10 20 - -
Flagellin - + + + + +
E
IL-1β
iNOS
GAPDH
mTIDM (μM) - - - - 10 20
wtTIDM (μM) - - 10 20 - -
CpG ODN - + + + + +
F
IL-1β
iNOS
GAPDH

Figure S6

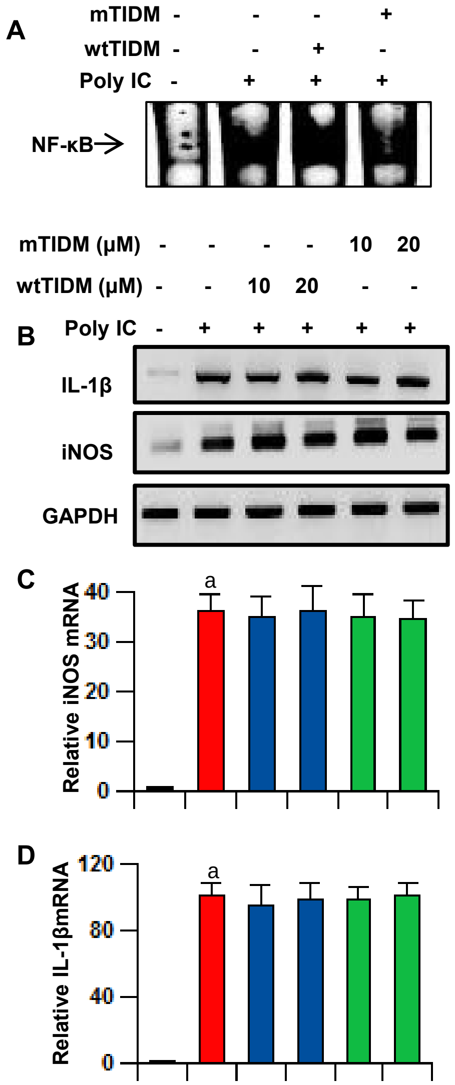
A
mTIDM - - - +
wtTIDM - - + -
Poly IC - + + +
NF-κB
mTIDM (μM) - - - - 10 20
wtTIDM (μM) - - 10 20 - -
B
Poly IC - + + + + +
IL-1β
iNOS
GAPDH
C
Relative iNOS mRNA
0
10
20
30
40
a
D
Relative IL-1βmRNA
0
40
80
120
a

Figure S7

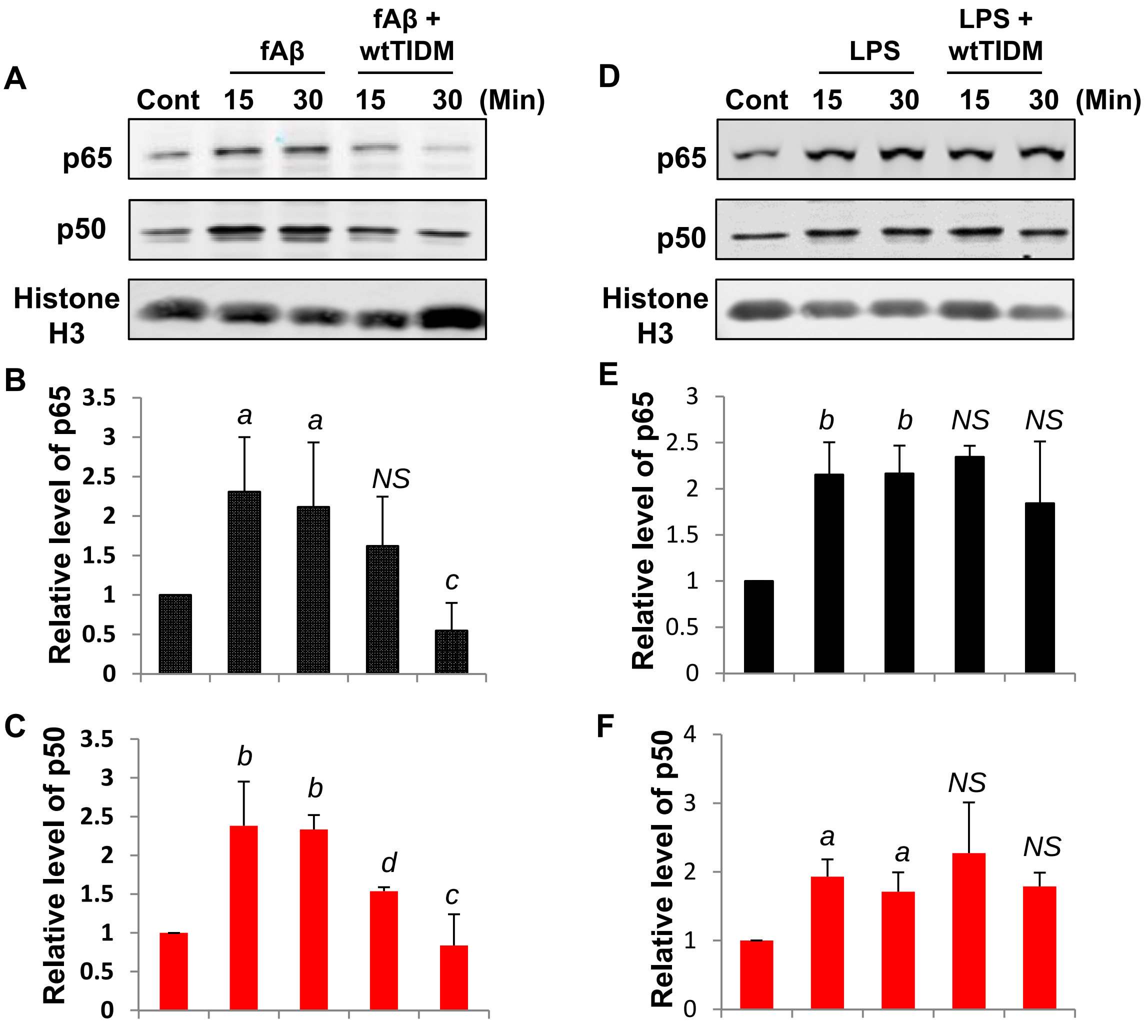
A
fAβ
fAβ + wtTIDM
Cont 15 30 15 30 (Min)
p65
p50
Histone H3
B
Relative level of p65
3.5
3
2.5
2
1.5
1
0.5
0
a
a
NS
c
C
Relative level of p50
b
b
d
c
D
LPS
LPS + wtTIDM
Cont 15 30 15 30 (Min)
p65
p50
Histone H3
E
Relative level of p65
b
b
NS
NS
F
Relative level of p50
4
3
2
1
0
a
a
NS
NS

Figure S8

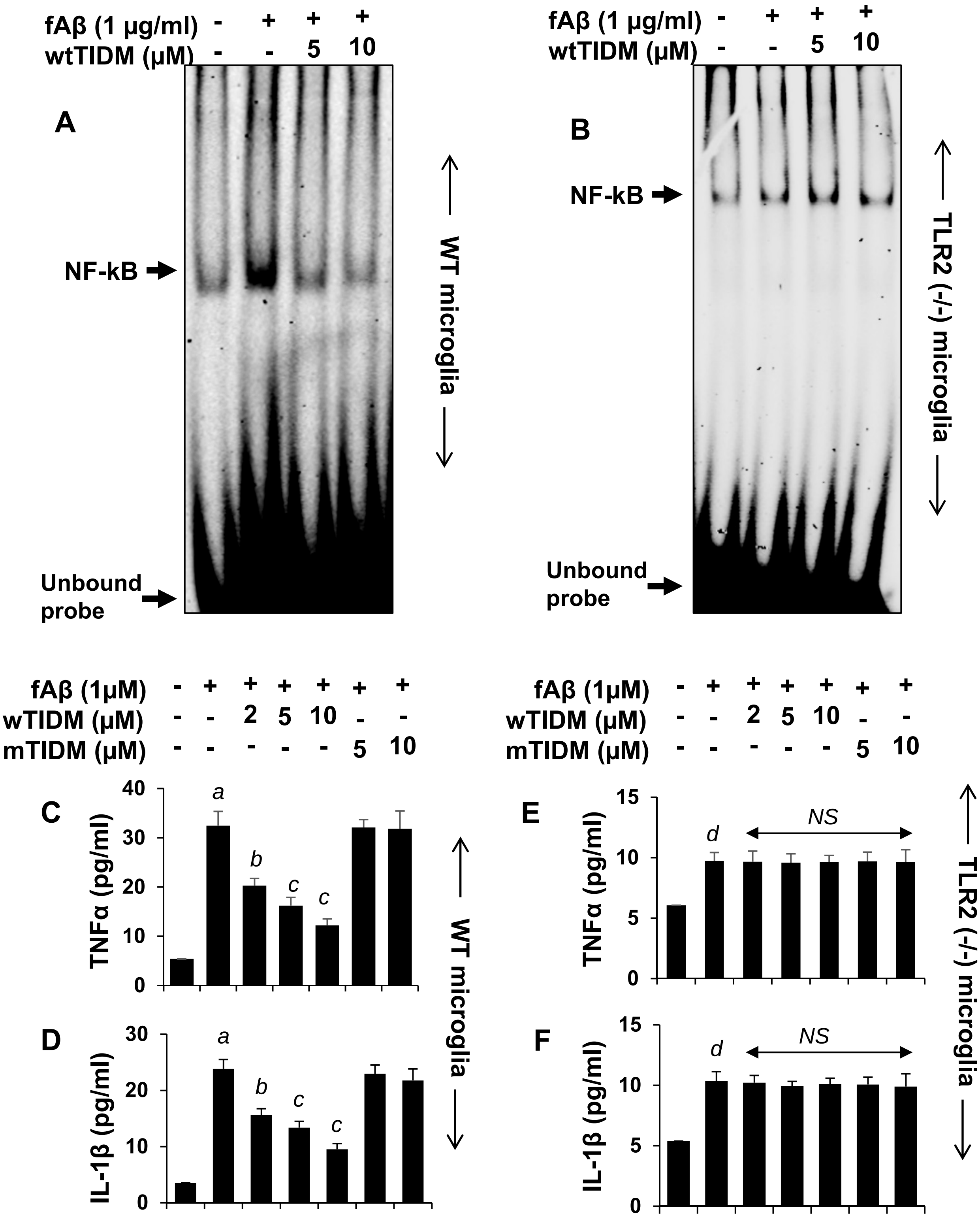

A
fAβ (1 µg/ml) - + + +
wtTIDM (µM) - - 5 10
NF-kB
Unbound probe
WT microglia
B
fAβ (1 µg/ml) - + + +
wtTIDM (µM) - - 5 10
NF-kB
Unbound probe
TLR2 (-/-) microglia
fAβ (1µM) - + + + + + +
wTIDM (µM) - - 2 5 10 - -
mTIDM (µM) - - - - - 5 10
C
TNFα (pg/ml)
D
IL-1β (pg/ml)
a
b
c
WT microglia
E
TNFα (pg/ml)
F
IL-1β (pg/ml)
d
NS
TLR2 (-/-) microglia

Figure S9

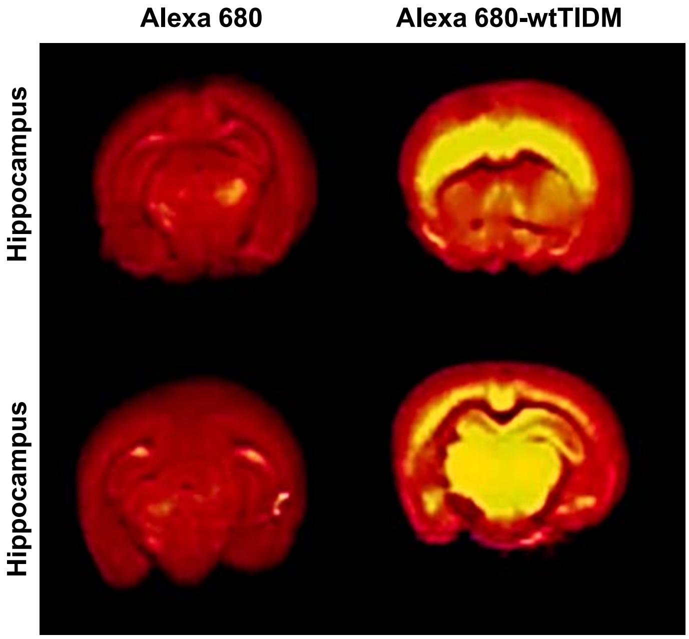

Figure S10

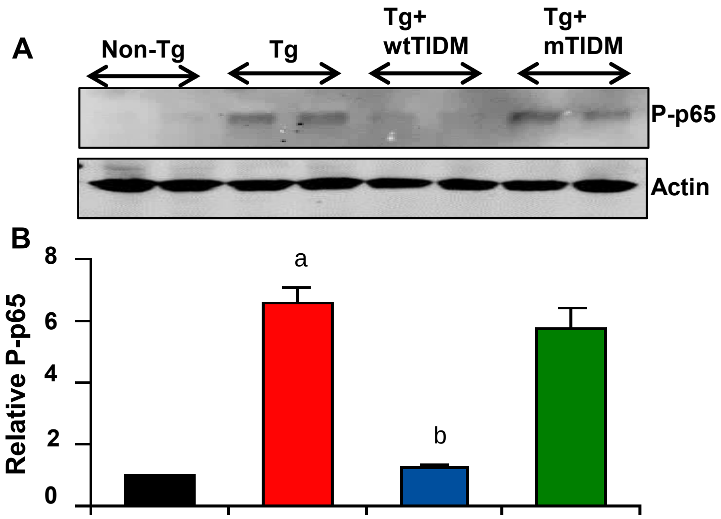
A
Non-Tg
Tg
Tg+
wtTIDM
Tg+
mTIDM
P-p65
Actin
B
Relative P-p65
8
6
4
2
0
a
b

Figure S11

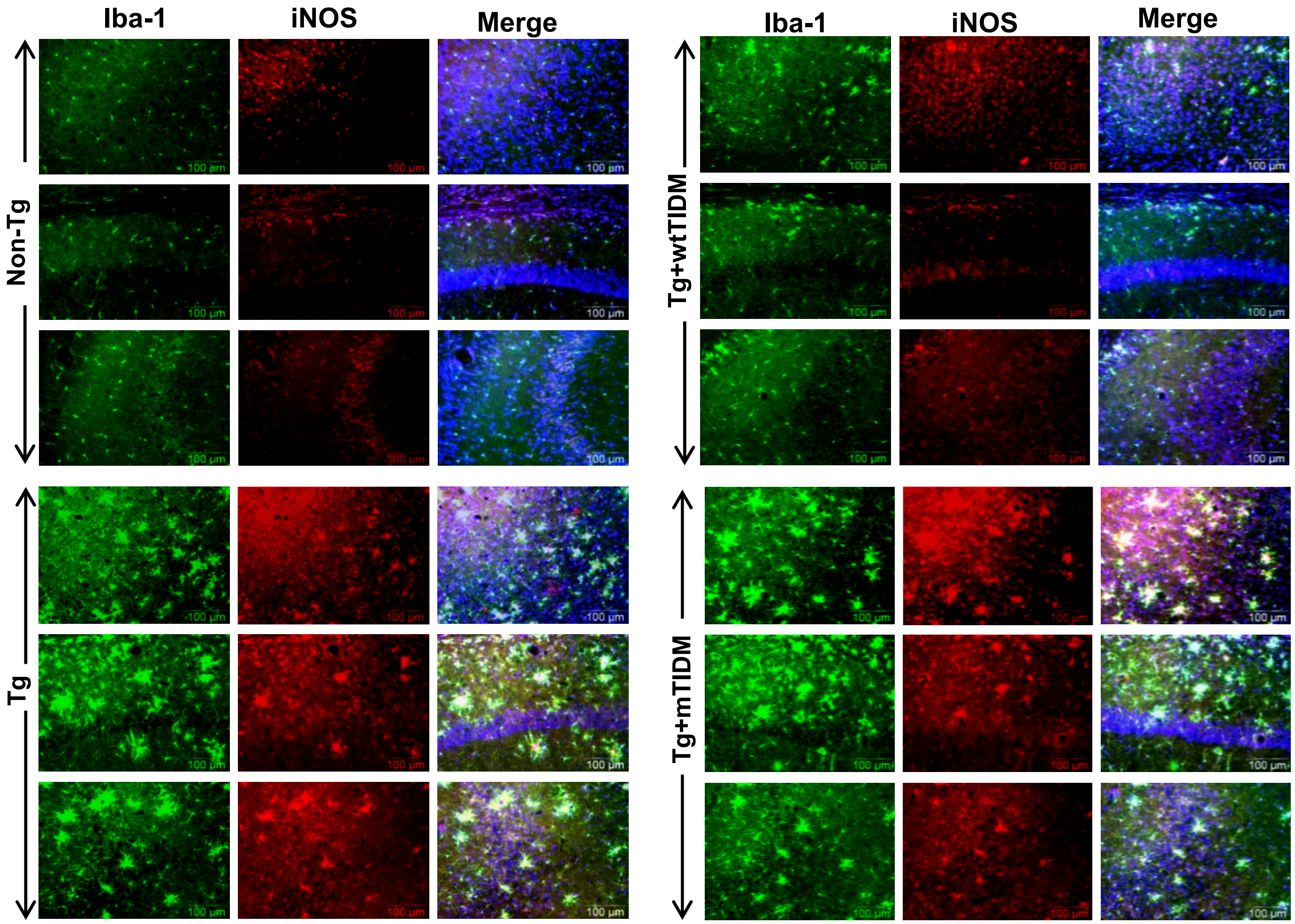
Iba-1
iNOS
Merge
Iba-1
iNOS
Merge
Non-Tg
Tg
Tg+wtTIDM
Tg+mTIDM
100 µm

Figure S12

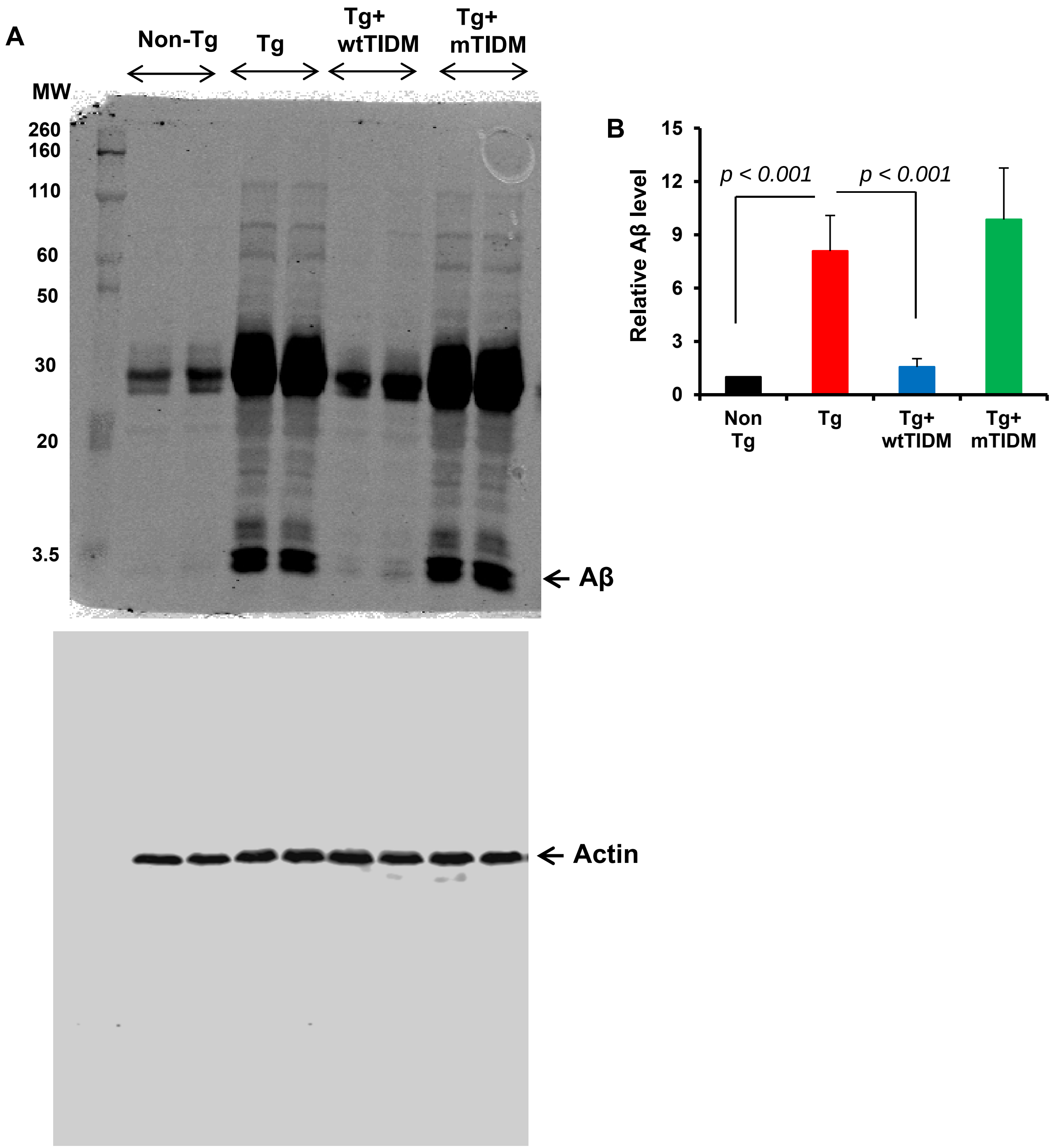
A
Non-Tg
Tg
Tg+ wtTIDM
Tg+ mTIDM
MW
260
160
110
60
50
30
20
3.5
Aβ
Actin
B
15
12
9
6
3
0
Relative Aβ level
p < 0.001
p < 0.001
Non Tg
Tg
Tg+ wtTIDM
Tg+ mTIDM

Figure S13

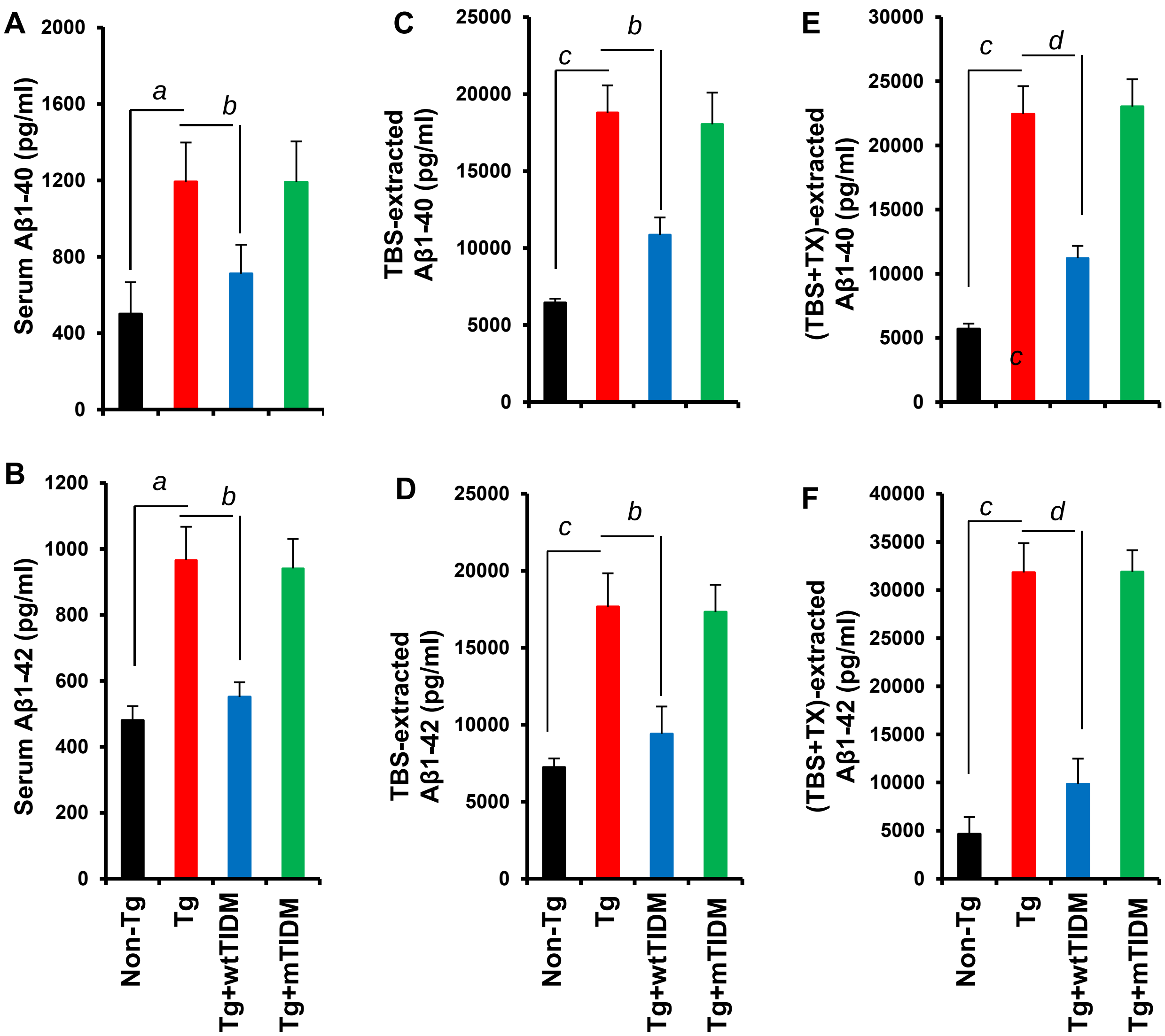
A
B
C
D
E
F
Serum Aβ1-40 (pg/ml)
Serum Aβ1-42 (pg/ml)
TBS-extracted Aβ1-40 (pg/ml)
TBS-extracted Aβ1-42 (pg/ml)
(TBS+TX)-extracted Aβ1-40 (pg/ml)
(TBS+TX)-extracted Aβ1-42 (pg/ml)
a
b
c
d
Non-Tg
Tg
Tg+wtTIDM
Tg+mTIDM

Figure S14

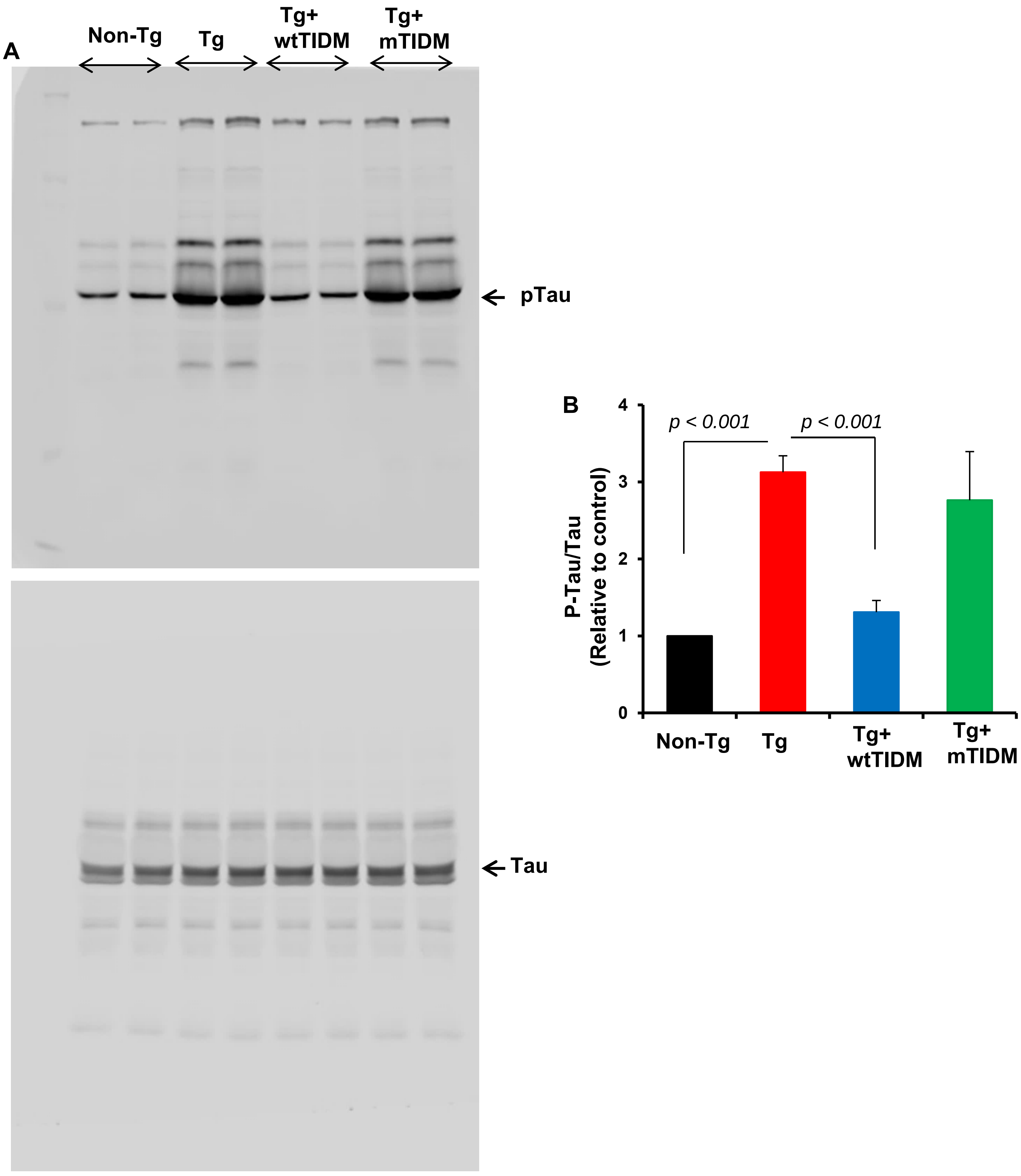
A
Non-Tg
Tg
Tg+ wtTIDM
Tg+ mTIDM
pTau
Tau
B
p < 0.001
p < 0.001
P-Tau/Tau (Relative to control)
0
1
2
3
4
Non-Tg
Tg
Tg+ wtTIDM
Tg+ mTIDM

Figure S15

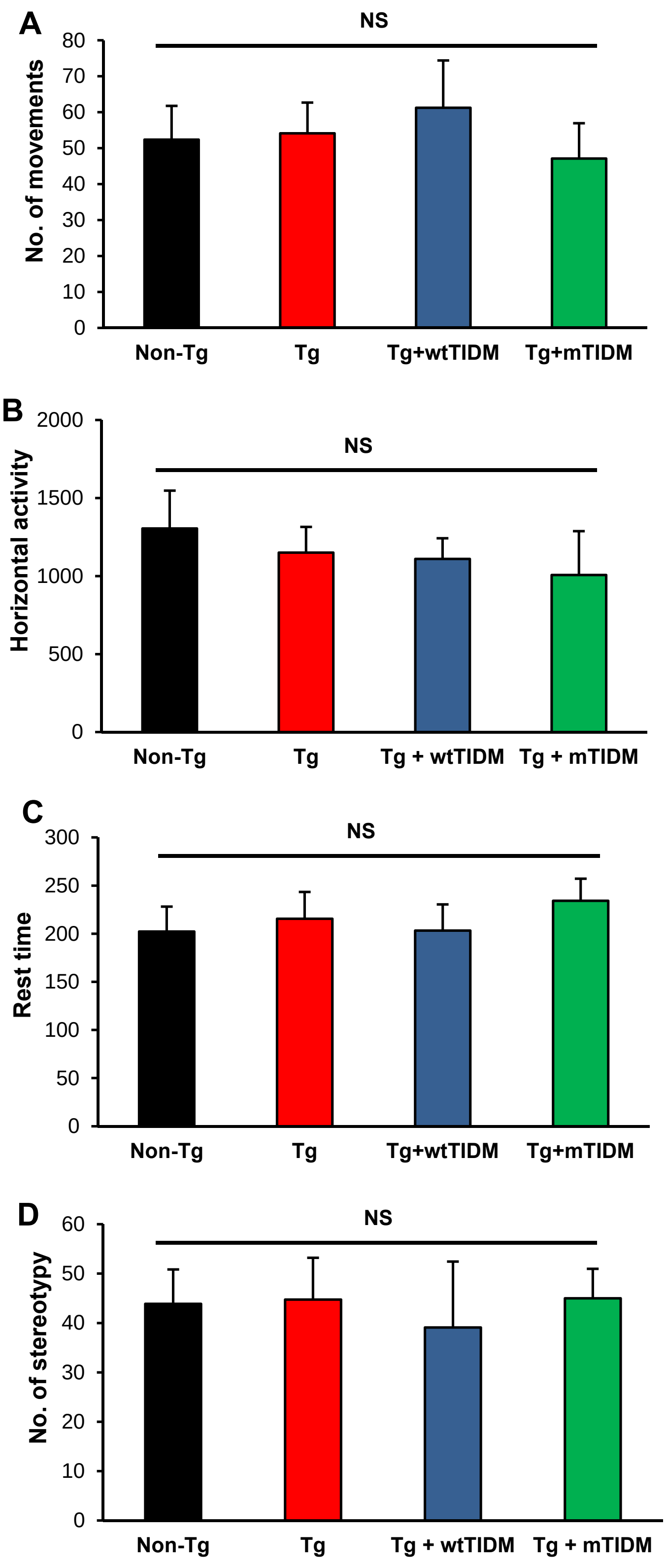
A
NS
No. of movements
0
10
20
30
40
50
60
70
80
Non-Tg
Tg
Tg+wtTIDM
Tg+mTIDM
B
NS
Horizontal activity
0
500
1000
1500
2000
Non-Tg
Tg
Tg + wtTIDM
Tg + mTIDM
C
NS
Rest time
0
50
100
150
200
250
300
Non-Tg
Tg
Tg+wtTIDM
Tg+mTIDM
D
NS
No. of stereotypy
0
10
20
30
40
50
60
Non-Tg
Tg
Tg + wtTIDM
Tg + mTIDM

Figure S16

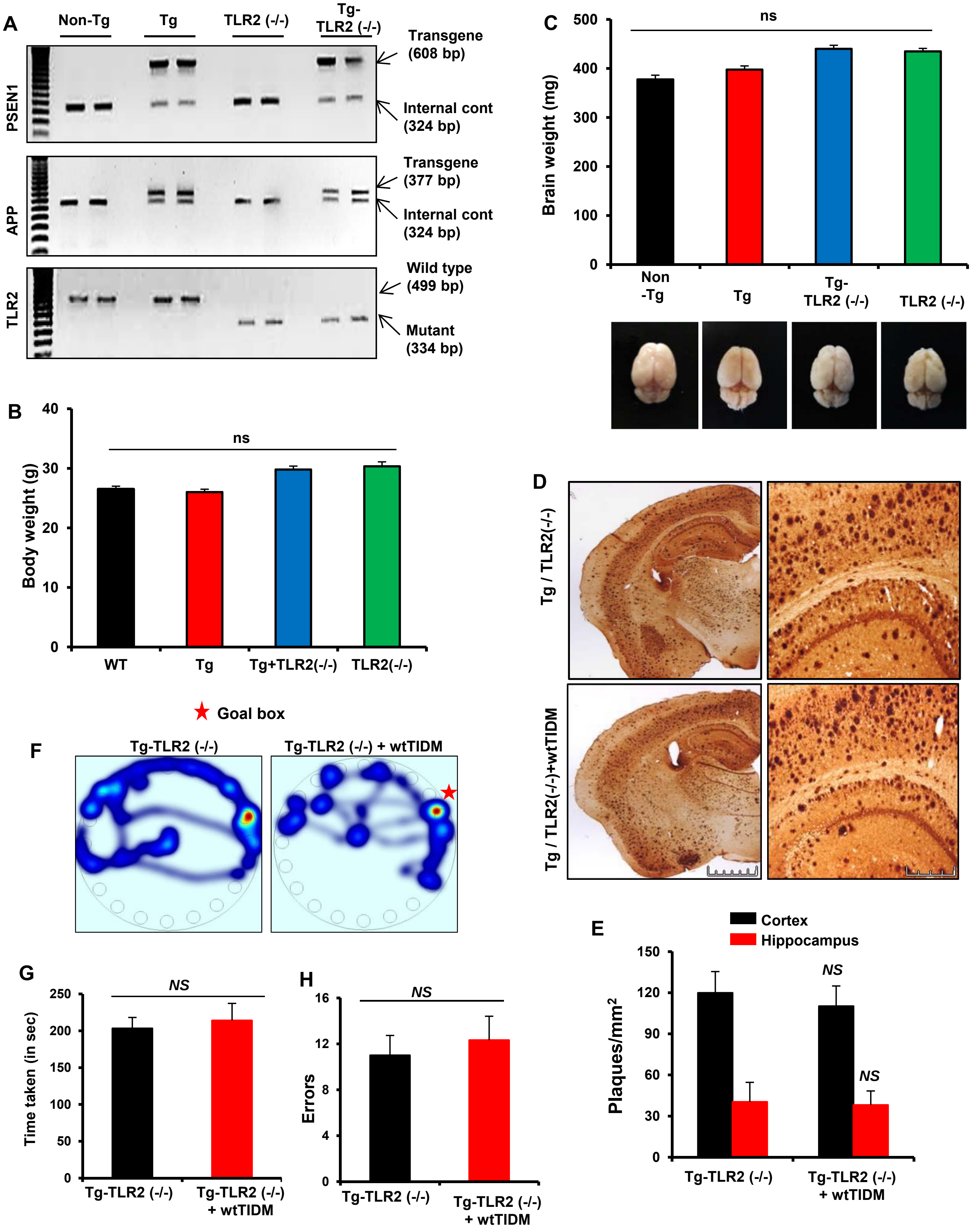
A
Non-Tg
Tg
TLR2 (-/-)
Tg-
TLR2 (-/-)
PSEN1
Transgene
(608 bp)
Internal cont
(324 bp)
APP
Transgene
(377 bp)
Internal cont
(324 bp)
TLR2
Wild type
(499 bp)
Mutant
(334 bp)
C
ns
Brain weight (mg)
500
400
300
200
100
0
Non
-Tg
Tg
Tg-
TLR2 (-/-)
TLR2 (-/-)
B
ns
Body weight (g)
40
30
20
10
0
WT
Tg
Tg+TLR2(-/-)
TLR2(-/-)
D
Tg / TLR2(-/-)
Tg / TLR2(-/-)+wtTIDM
Goal box
F
Tg-TLR2 (-/-)
Tg-TLR2 (-/-) + wtTIDM
E
Cortex
Hippocampus
Plaques/mm2
150
120
90
60
30
0
NS
NS
Tg-TLR2 (-/-)
Tg-TLR2 (-/-)
+ wtTIDM
G
NS
Time taken (in sec)
250
200
150
100
50
0
Tg-TLR2 (-/-)
Tg-TLR2 (-/-)
+ wtTIDM
H
NS
Errors
16
12
8
4
0
Tg-TLR2 (-/-)
Tg-TLR2 (-/-)
+ wtTIDM

Figure S17

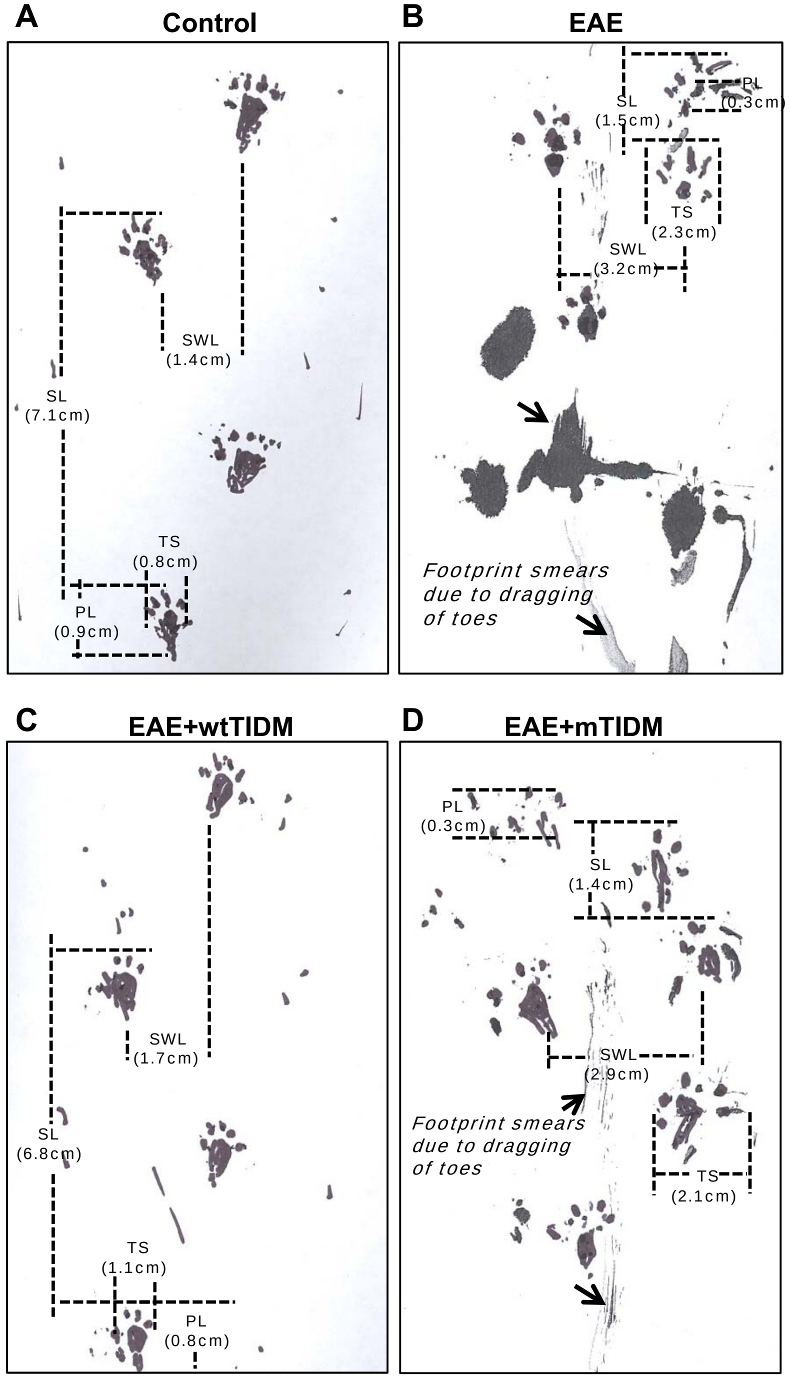

SL, Stride length; SWL, Sway length; PL, Print length; TS, Toe spread

Figure S18

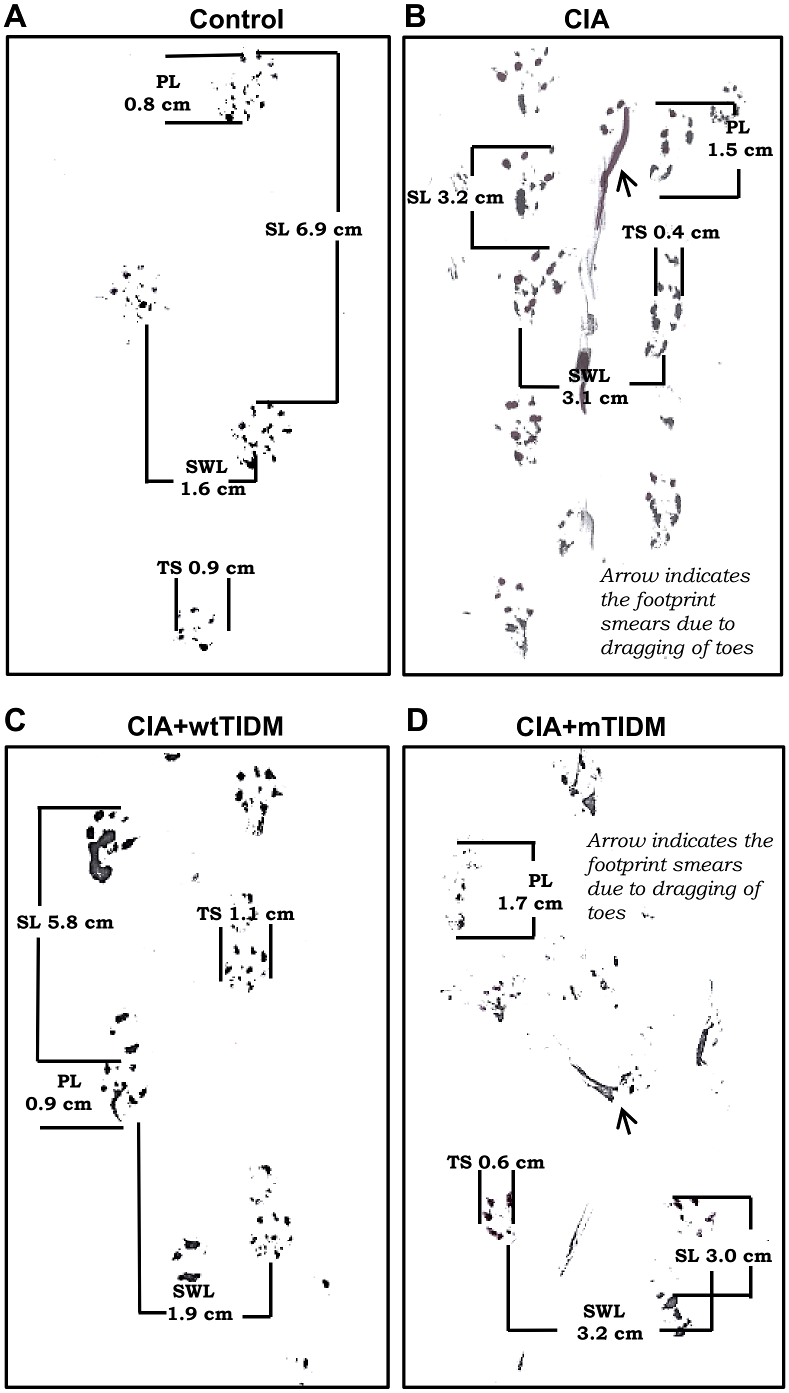

SL, Stride length; SWL, Sway length; PL, Print length; TS, Toe spread

Figure S19

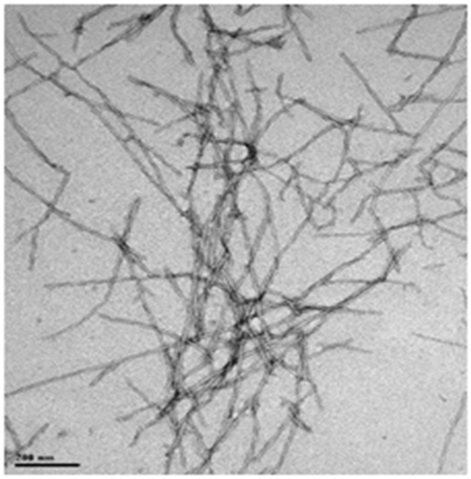

Figure S20
